# The Close Interaction of a C−F Bond with an Amide Carbonyl: Crystallographic and Spectroscopic Characterization

**DOI:** 10.1002/anie.202207966

**Published:** 2022-07-13

**Authors:** Stefan Andrew Harry, Muhammad Kazim, Phuong Minh Nguyen, Andrea Zhu, Michael Richard Xiang, Jonathan Catazaro, Maxime Siegler, Thomas Lectka

**Affiliations:** ^1^ Department of Chemistry Johns Hopkins University 3400 N. Charles St. Baltimore MD 21218 USA

**Keywords:** Amides, Fluorine, Non-Covalent Interactions

## Abstract

The putative interaction of a C−F bond with an amide carbonyl has been an intriguing topic of interest in this century for reasons spanning basic physical organic chemistry to biochemistry. However, to date, there exist no examples of a close, well‐defined interaction in which its unique aspects can be identified and exploited. Herein, we finally present an engineered system possessing an exceptionally tight C−F‐amide interaction, allowing us to obtain spectroscopic, crystallographic, and kinetic details of a distinctive, biochemically relevant chemical system for the first time. In turn, we also explore Lewis acid coordination, C−F bond promotion of amide isomerization, enantiomerization, and ion protonation processes.

## Graphical abstract

An engineered system possessing an exceptionally tight C−F‐amide interaction is presented, which allows spectroscopic, crystallographic, and kinetic details of a distinctive, biochemically relevant chemical system to be obtained for the first time. The Lewis acid coordination, C−F bond promotion of amide isomerization, enantiomerization, and ion protonation processes are explored.

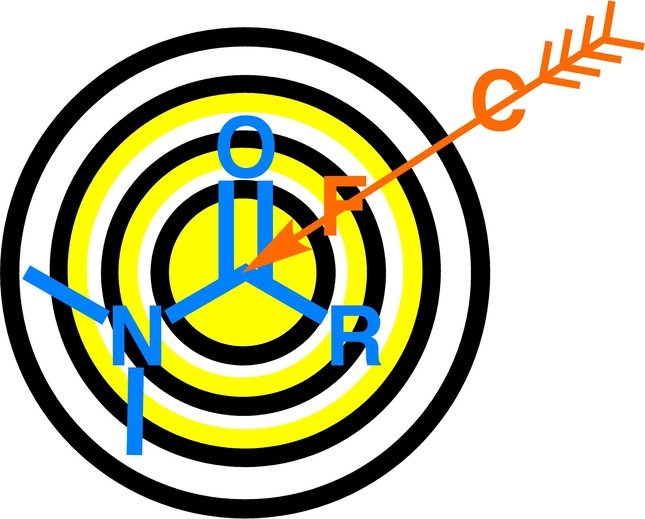

The days are long past when the fluorine of a C−F bond has been thought of as merely an inert hydrogen atom surrogate in molecules of biological interest.^[1]^ Although the C−F bond can indeed stabilize classical hydrophobic arrangements through favorable dispersion forces, recent research instead has unveiled fluorine‘s ability to engage in hydrogen bonding, in particular to amide N−H groups and acidic side chains in proteins.^[2]^ Additionally, C−F bonds can interact, albeit weakly, with the carbonyl carbon atoms of amide groups in putative “n→π*”^[3]^ contacts (Figure 1).^[4]^ The C−F amide interaction, while observable in crystals, is otherwise difficult to characterize spectroscopically. What were to happen instead if a *forced* geometry were to bring the interacting partners into close, inflexible proximity?^[5]^ A weak, loose interaction^[6]^ thereby becomes substantial, a subtle trend turns prominent, and an anomaly unveils a unique pattern. In this communication, we present a rigid, idealized model system based on a 4,5‐disubstituted “cross‐bay” phenanthrene^[7]^ to afford an extraordinarily close C−F‐amide contact. This allows us to probe the C−F amide interaction in a clear and convincing way through NMR, X‐ray, IR, UV, computational, and mass spectral studies.

**Figure 1:**
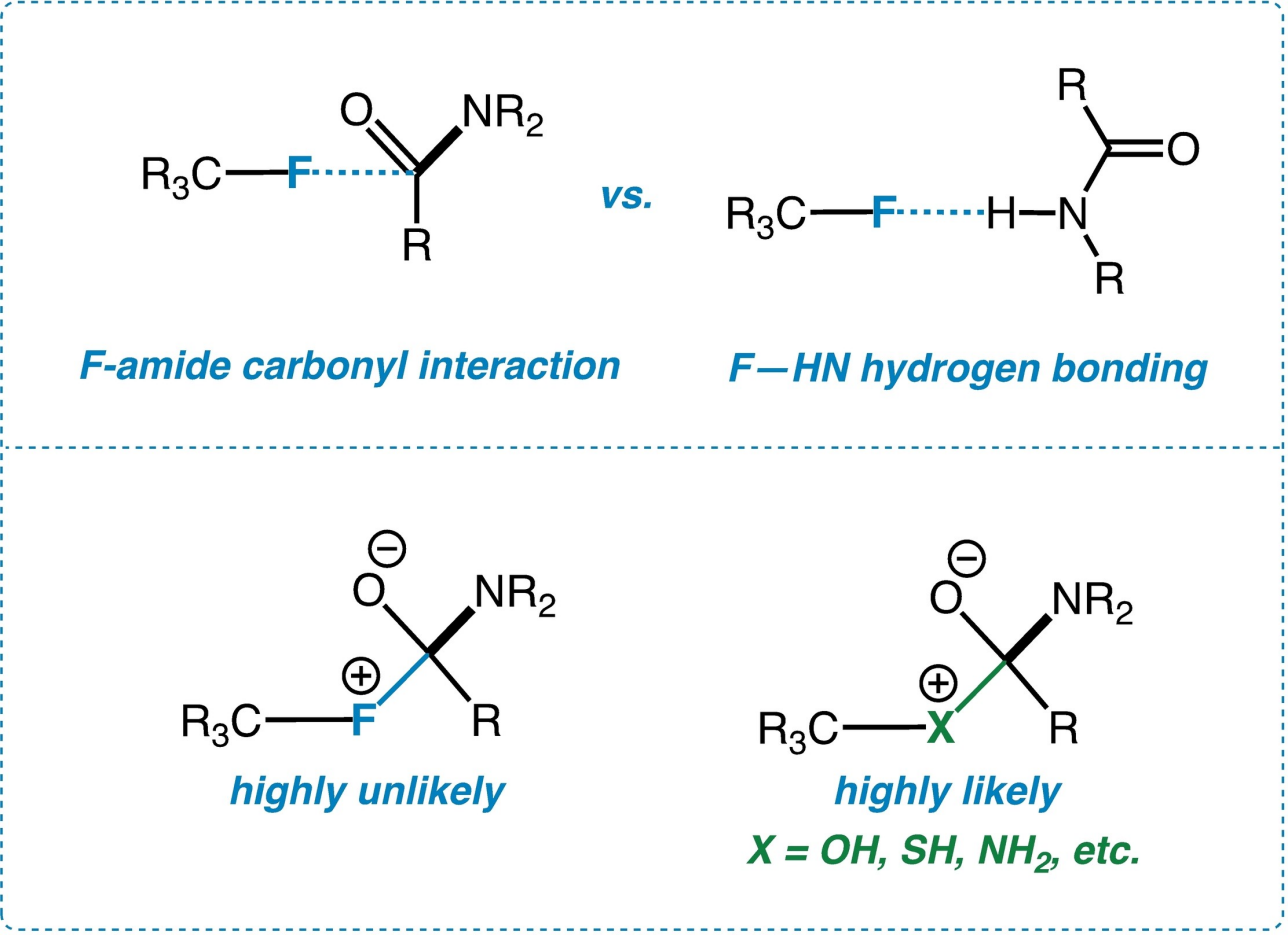
Fluorine‐amide carbonyl interaction.

A simple survey within the CSD^[8]^ and PBD databases^[9]^ shows a potential abundance of such interactions in crystal structures of small, fluorinated molecules. These examples include both intramolecular and intermolecular interactions with various bound ions or hosts. Several years ago, Diederich et al. published a highly informative study outlining the basic spatial characteristics of C−F‐amide carbonyl contacts in proteins and related inhibitor complexes highlighting their importance.^[10]^ Figure 2 shows an array of close contacts in crystal structures deposited in the CSD as of 2022, revealing a variety of C−F orientations. Clusters of C−F bonds are seen to interact with amide carbonyl groups within the van der Waals radii of carbon and fluorine; clustering is especially prominent in aromatic‐amide—aliphatic‐F arrays, a fact which we explicitly address in our system. Diederich et al. also explored the energetic consequences of interactions between CF_3_ and Ar−F groups with an amido group in Wilcox‐type molecular torsion balances,^[11]^ although given the flexibility of the model systems, the precise origins of the interactions remain unclear. Possibilities include C−F‐amide carbonyl contacts, N−H—O=C hydrogen bonding, π‐stacking, and/or the participation of a dipolar ArH—O=C interaction.^[12]^ Very recently, a tenuous claim of a C−F‐amide interaction in a model system is instead best explained as an example of NMR spectroscopic diastereotopicity.^[13]^

**Figure 2:**
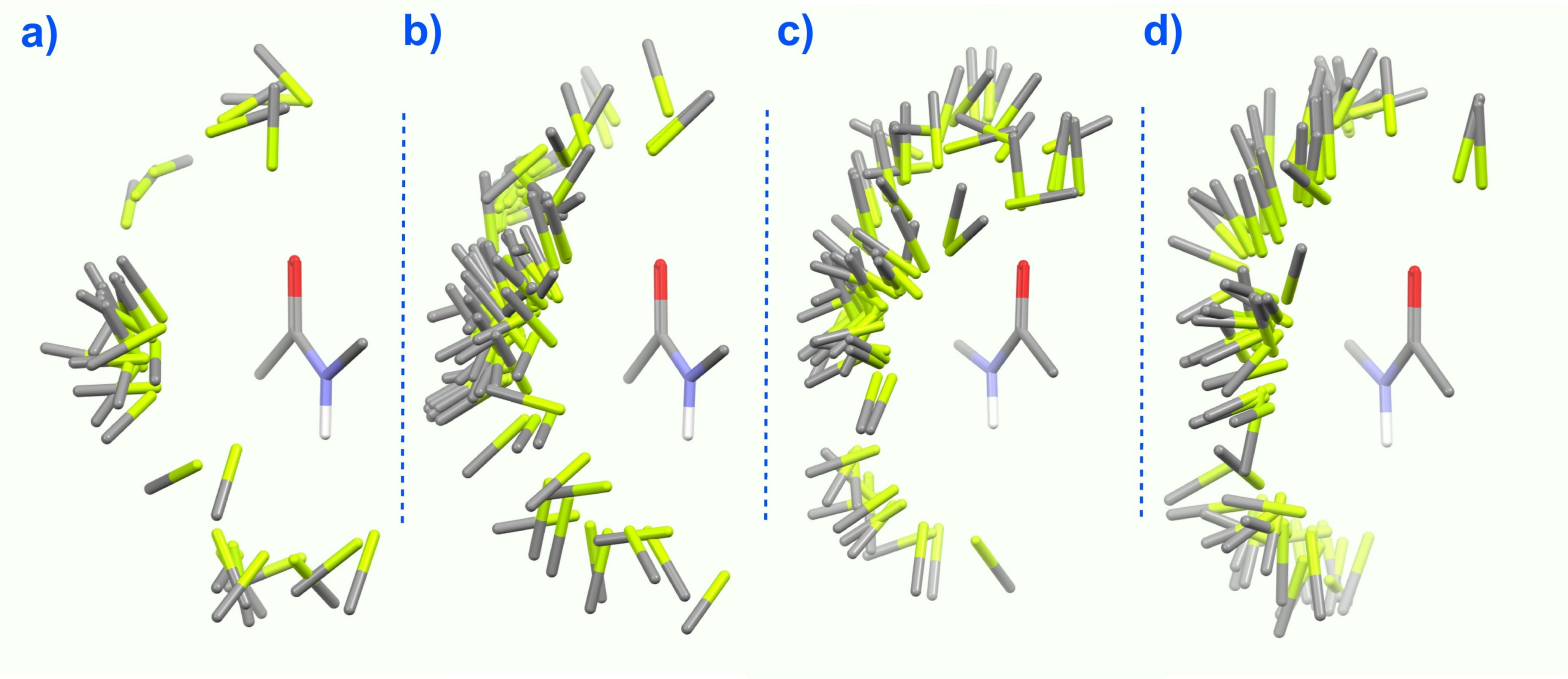
C−F bond—amide carbonyl interactions from crystal structures in the CCBD within van der Waals radii of C and F (F=green, C=gray, O=red, N=blue); Isostar program, CCDB. a) aliphaticCON(aromatic). b) aromaticCON(aliphatic). c) aromaticCON(aromatic). d) aliphaticCON‐(aliphatic).

The C−F—amide carbonyl interaction is relevant for another reason. Although nucleophilic in character, it is highly unlikely that the fluorine of a C−F bond will form a classical tetrahedral intermediate (essentially a C−F−C fluoronium ion^[14]^) with the amide functional group (Figure 1). In a sense, the fluorine in a C−F bond can be thought of as an interrupted or “frozen” nucleophile. Unlike other heteroatoms containing lone pairs of electrons, fluorine is unlikely to initiate covalent bond formation. This “snapshot in place” could provide valuable insights into fluorine‘s role in macromolecular stabilization without presenting a danger of precocious reactivity. Along the way, our study also addresses analogous interactions of C−F bonds with ester and aldehyde groups as comparisons and controls.

Our synthetic scheme began with the Wittig reaction (Figure 3) of 3,5‐difluorobenzaldehyde and dimethyl 5‐(triphenylphosphinomethyl)isophthalate **1**.^[15]^ The intermediate stilbene (a mixture of *cis* and *trans* isomers) was subjected to saponification to form the dicarboxylic acid; treatment with excess thionyl chloride in CH_2_Cl_2_ afforded diacid chloride **2**. Oxidative photochemical Mallory cyclization^[16]^ (254 nm, I_2_, MeCN) followed by amidation (HNMe_2_, CH_2_Cl_2_) provides the target diamide **3** and control diester **4** as crystalline white solids. The electron withdrawing nature of the acyl chloride subdued side reactions to ensure success. Control dialdehyde **7** requires a different pathway for its construction: 1) Wittig reaction with 3,5‐dimethylbenzyl phosphonium bromide **5** and 3,5‐difluorobenzaldehyde, 2) followed by oxidative photochemical cyclization, 3) NBS bromination, 4) substitution with silver nitrate, 5) hydrogenation to the diol, and 6) ultimately, oxidation by PCC.

**Figure 3:**
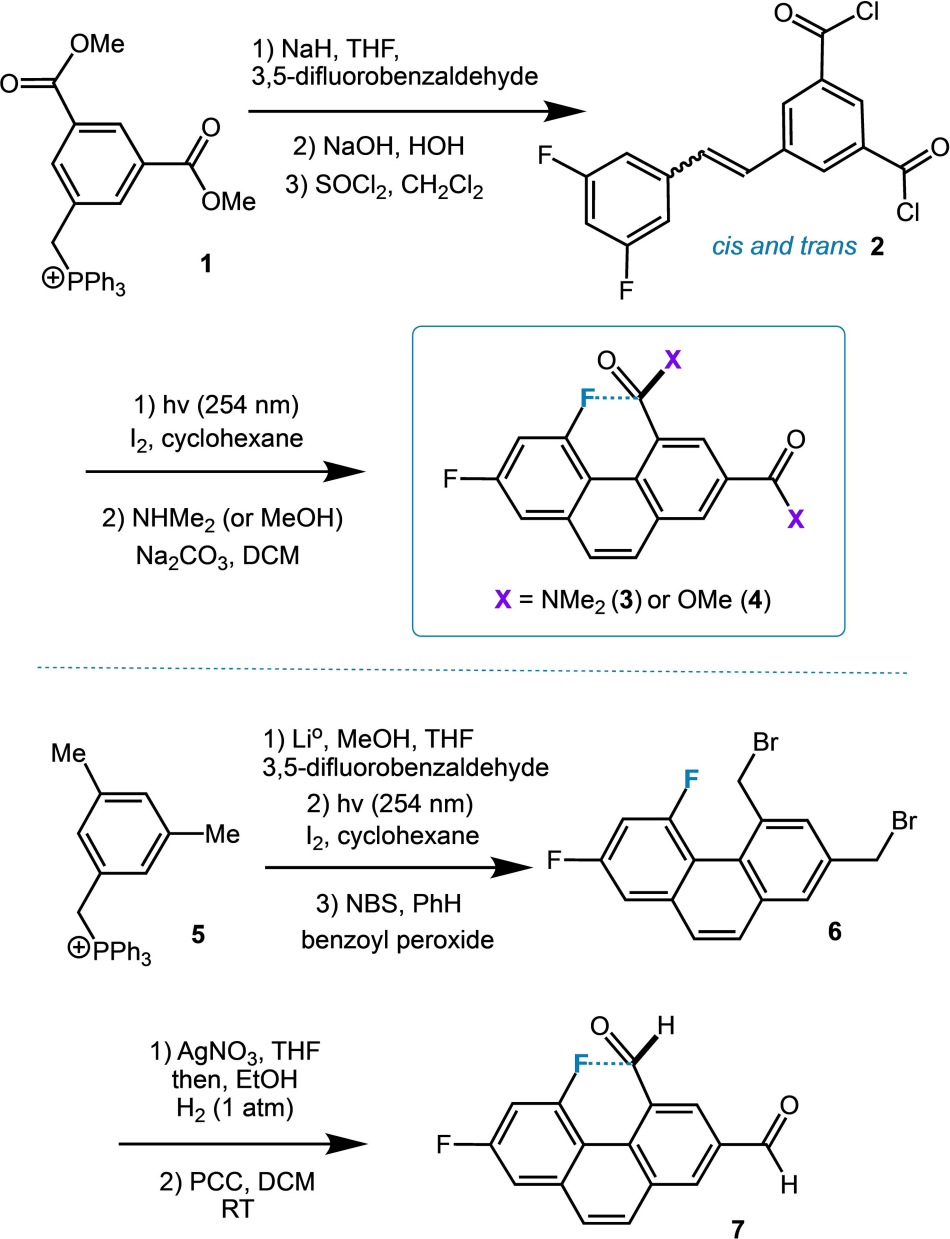
Synthetic routes to **3**, **4** and **7**.

With diamide **3** in hand, we turned to X‐ray crystallography to identify several significant interactions. First, its crystal structure (Figure 4) shows an F—C=O distance of 2.448(15) Å. This distance (best reproduced at M062X/6‐311++G** as 2.46 Å) is about 0.2 Å shorter than the next closest measured F—C=O interaction, which relies on intermolecular ionic crystal packing (a carbonyl‐silver(I) secondary coordination),[^8^, ^17a^] and 0.33 Å shorter than the distance reported for a more relevant system in Paulini's review.^[17b]^ The measured F—C=O (F1‐C18‐O2) angle is 95.1°, and the torsional aromatic distortion (C1−C2−C4−C5) is −17.64° (reproduced at M062X/6‐311++G** as 95.6° and −19.7°; see Supporting Information page S28 for expanded image of the crystal packing). Furthermore, suitably large, high‐quality crystals were used to determine an electron static deformation density map,^[18]^ giving the viewer a very close look at the nature of the interaction. Most notably, the electron‐deficient region behind the carbonyl carbon is located next to a high‐density region due to fluorine's lone pair (Figure 5a–c). Based on the contour diagram, it is geometrically configured to allow an optimal n→π* interaction that is clearly electrostatic in origin, with little covalent character. Computationally, this observation is confirmed by an NBO analysis of the electron density between F and the carbonyl carbon of **3**, which reveals an electron deficient trough that takes the form of a saddle point (Figure 5d, also see Supporting Information page S33). The anomalous electron distribution surrounding the probe amide nitrogen atom (labeled N2) is consistent with its evident pyramidalization (improper torsion angle C18−N2−C19−C20=159.7°; control amide group 171.1°). Moreover, an atoms‐in‐molecules (AIM) analysis shows a bond critical point^[19]^ with an electron density of 0.0213 e Å^−3^ between the carbonyl carbon of the probe amide group and the fluorine atom, indicative of an electrostatic interaction.^[20]^

**Figure 4:**
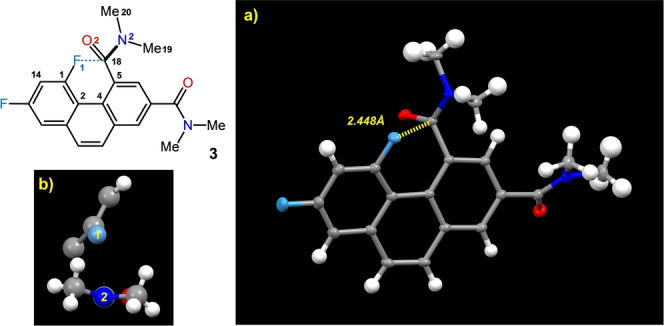
a) X‐ray crystal structure of diamide **3** (50 % thermal ellipsoids). b) Cut‐away of probe region revealing amide pyramidalization and the angle of distortion (improper torsion C18−N2−C19−C20 159.73°).

**Figure 5:**
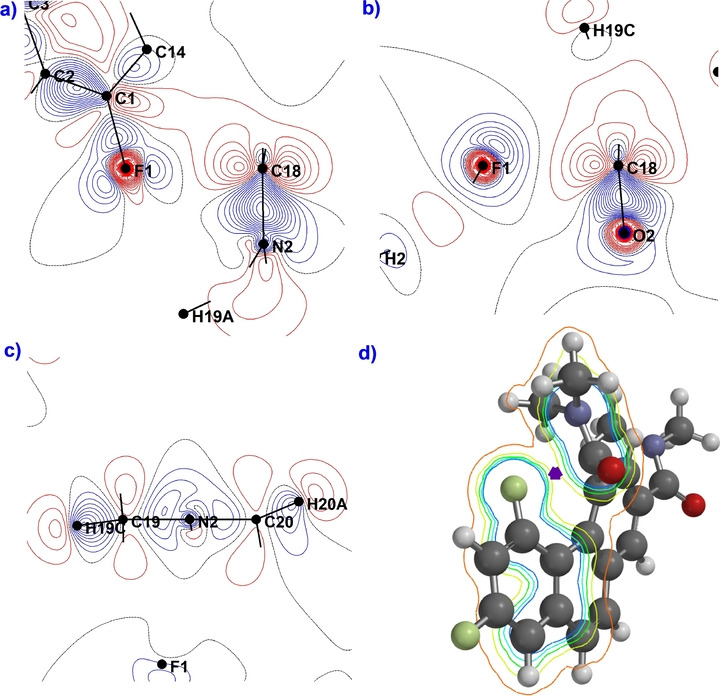
Contour slices of the static deformation density map in the vicinity of the F‐amide region of **3** (positive (blue) and negative (red) contours are drawn at intervals 0.05 e Å^−3^, black=0.00 e Å^−3^). a) Primarily electrostatic interaction along the F−carbonyl axis, (C18 denotes the probe carbonyl carbon). b) Alternate view including the C−O axis. c) Anomalous electron density surrounding the pyramidalized probe amide nitrogen. d) NBO‐derived electron density contour slice calculated at M062X/6‐311++G** (0.04 isovalue) of **3** bisecting the axis of the C−F—C=O interaction (Spartan Program). The purple polygon marks an effective saddle point.

Interestingly, in the crystal structure of the corresponding diester **4**, the C−F—carbonyl distance is 0.02 Å *longer* than amide **3** (reproduced by M06 calc.). The electrostatic potential surface maps (calcd. at M06, Figure 6) provide a clue as to why. The map of diamide **3** in the probe region (a) shows the C−F bond contending with a region of positive electrostatic potential; the amide nitrogen's slight pyramidalization serves to move lone pair density away from fluorine. In diester **4** (b) the probe fluorine is close to an area of high negative potential. Rehybridization does not effectively alleviate this repulsion as the ether oxygen possesses two lone pairs of electrons.

**Figure 6:**
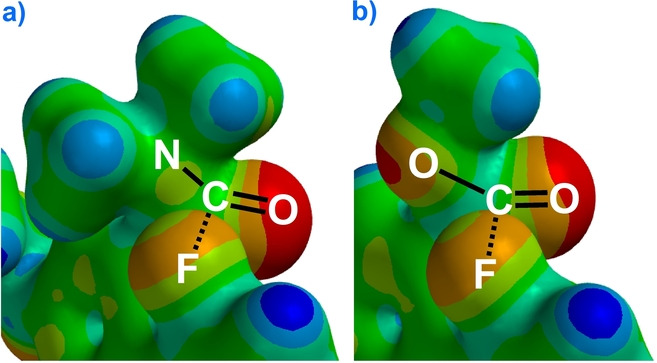
Electrostatic potential surface maps of the probe regions calculated at M062X/6‐311++G** (0.02 e au^−3^; property range −215 to 515 kJ); red negative; blue positive. a) Diamide **3**. b) Diester **4**.

Further proof was obtained through NMR spectroscopy of the carbonyl series (X=H, OMe, NMe_2_). All the ^13^C NMR spectra exhibit a diagnostic through‐space F—C=O coupling, indicative of the spatial proximity of the C−F bond and amide carbonyl.^[4]^ The magnitude of the coupling is largest for dialdehyde **7** (15.7 Hz; calc. M06 19.3 Hz) and smaller for amide **3** (6.3 Hz; calc. 7.2 Hz). However, diester **4** provides the smallest coupling (3.7 Hz; calc. 2.9 Hz); this may be explained once again through electrostatic mapping, electron‐electron repulsion, and F—C=O distances. DFT‐based NMR shielding calculations^[21]^ (M06) also mirror these results. The IR spectrum of **3** reveals two red‐shifted amide carbonyl stretches of 1602 cm^−1^ (red shift 32 cm^−1^) and 1620 cm^−1^ (red shift 14 cm^−1^ compared to control **8** ^[22]^ (see Figure 8) at 1634 cm^−1^; aryl protons instead of fluorine atoms), consistent with a close fluorine interaction. The amide groups of **3** combine to present heavily coupled vibrations;^[23]^ considering them independently is inadvisable. Finally, the UV spectrum of **3** also reveals a slight bathochromic shift in line with distortion of the aromatic system and amide functional groups.

The C−F—N_(amide)_ interaction should strengthen in the presence of Lewis acids. As a test case, we chose BBr_3_ for its pronounced ability to bind to amide carbonyls in a single point interaction.^[24]^ Treatment of a solution of **3** in CDCl_3_ with excess BBr_3_ (see Figure 7 to ensure limiting spectroscopic behavior) produces both dramatic *upfield* and *downfield* changes in the chemical shifts of the probe and control fluorine atoms respectively (−4.0 ppm and +5.3 ppm, Figure S10). Moreover, the ^13^C signal associated with the probe amide carbonyl carbon shows a coupling constant of 6.3 Hz in the absence of BBr_3_, while the treated solution reduces the coupling to 3.0 Hz. This observation, as well as the ^19^F signal shifts, are directly aligned with our calculated structures, chemical shifts, and coupling constants (M06); the optimized geometry of the doubly BBr_3_ coordinated moiety displays a closer C−F—N_(amide)_ distance by 0.03 Å (2.41 Å) when compared to free **3**. On the other hand, the computed partial negative charge on the probe fluorine decreases upon complexation, demonstrating once again that ^19^F shifts correlate poorly with this property^[25]^ (NBO charges: probe F=−0.372 a.u. whereas amide‐(BBr_3_)_2_ probe F=−0.353 a.u.).[^14b^, ^19^]

**Figure 7:**
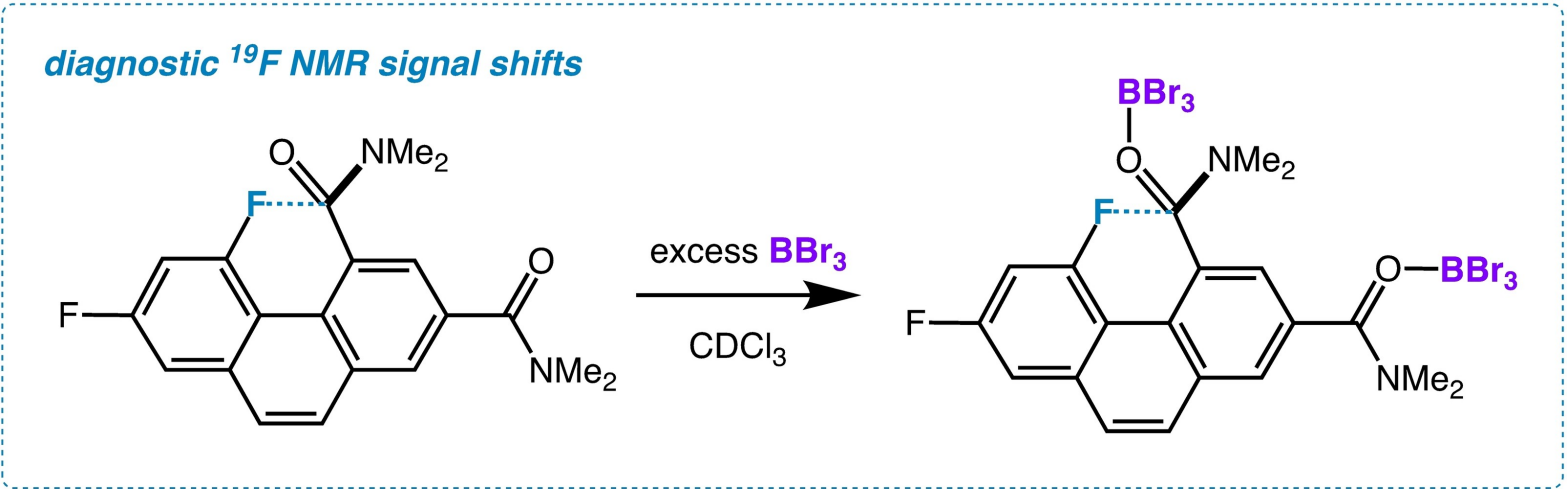
Lewis acid complexation of diamide **3**.

Amide isomerization about C−N bonds (AI) is a perennially intriguing topic in both biochemistry and physical organic chemistry. It can be catalyzed or promoted in different ways—metal ions, rotamase enzymes, and hydrogen bonding are most notable.^[27]^ The rarest method involves nucleophilic catalysis/promotion, which relies on a covalent, tetrahedral intermediate in which amide resonance is greatly reduced.^[28]^ It occurred to us that a C−F bond could provide a complementary way of promoting AI—not through a formal tetrahedral intermediate, but resulting instead from disruption of amide resonance by a through‐space interaction of the amide carbonyl with a C−F bond. In fact, when we compare the barriers to rotation of diamide **3** and spatially similar control amide **8** (steric effects can exert profound upward influences on AI barriers;^[29]^ we find that the barrier to rotation (Δ*G*_act_) is 3.9 kcal less in **3** when measured by line‐shape analysis^[30]^ (trend mirrored in calc. at M06; energy difference ΔΔ*G*_act_=3.0 kcal mol).^[31]^ Our investigation utilized variable‐temperature ^1^H NMR, as shown in Figure 8; distinctly exposing peak coalescence for the probe amide methyl groups (identified by ^1^H‐^19^F HOESY).

**Figure 8:**
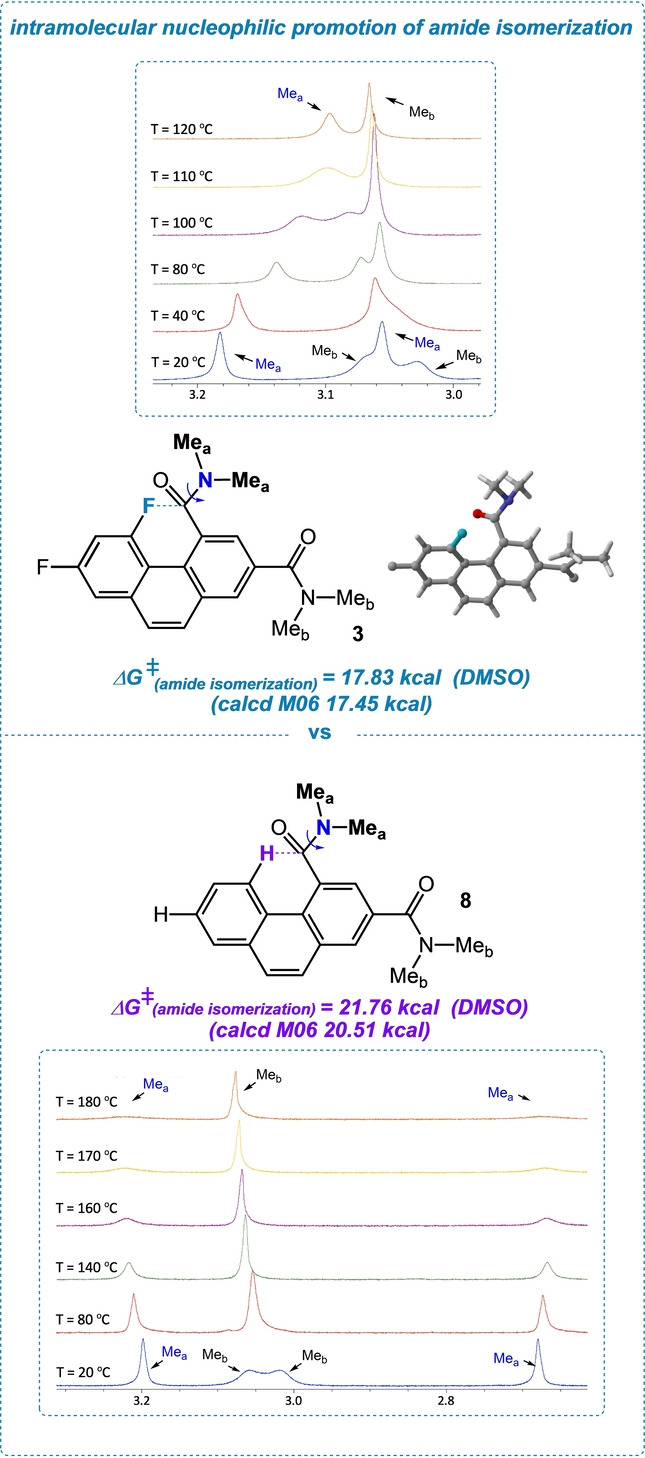
Calculated intramolecular nucleophilic promotion of amide isomerization (AI) in diamide **3**; variable‐temperature ^1^H NMR in DMSO.

Examination of a possible transition state geometry for AI reveals that it should lead to two structurally different products, even though the amide substituents are identical. Further discrete steps are necessary, theoretically posing the possibility that the rate‐determining step is not in fact AI per se. AI may be coupled to enantiomerization,^[32]^ whose dynamics can be probed through ^19^F NMR. In fact, diamide **3** crystallizes as a racemic mixture. We sought to differentiate the enantiomers in solution through a chiral shift reagent, assuming low rates of interconversion on the NMR time scale. When we treated a solution of **3** in CDCl_3_ with varying quantities of an Eu^III^ derivative of 3‐trifluoroacetyl‐*d*‐camphor [Eu(facam)_3_]^[33]^ at 0 °C, a partial splitting of the distal (control) fluorine atom into two doublets of identical intensity could be observed (the probe fluorine is severely broadened by paramagnetic effects).

Variable‐temperature ^1^H NMR revealed coalescence at 55°C (see Figure 10), permitting the calculation of an approximate Δ*G*_act_=17.1 kcal mol^−1^. This result is very close to the measured barrier for AI; the difference may be attributable to a solvent effect on going from CDCl_3_ to d_6_‐DMSO. The enantiomerization of **3** is a complex process involving *three* putative transition states. The Δ*G*_act_ reflects rate‐determining simultaneous rotations about C−C and C−N amide bonds that surmount the steric impediment of an α‐hydrogen atom (TS1 −885.789313 Ha; M06 calcd. Δ*G*_act_ in good agreement at 17.5 kcal mol^−1^ ^[26]^). Intermediate **3 a** is high energy and overcomes a steric clash between fluorine and oxygen to produce diastereomer **3 b**. Note that **3 a** and TS2 are linked by a very small, albeit extant barrier. TS3 is the low energy transition state (capitalizing on a favorable interaction of the amide carbonyl with fluorine) that finally leads to the enantiomer (ent‐**3**, Figure 9 and 10). Thus, in our case, AI and enantiomerization are synonymous.

**Figure 9:**
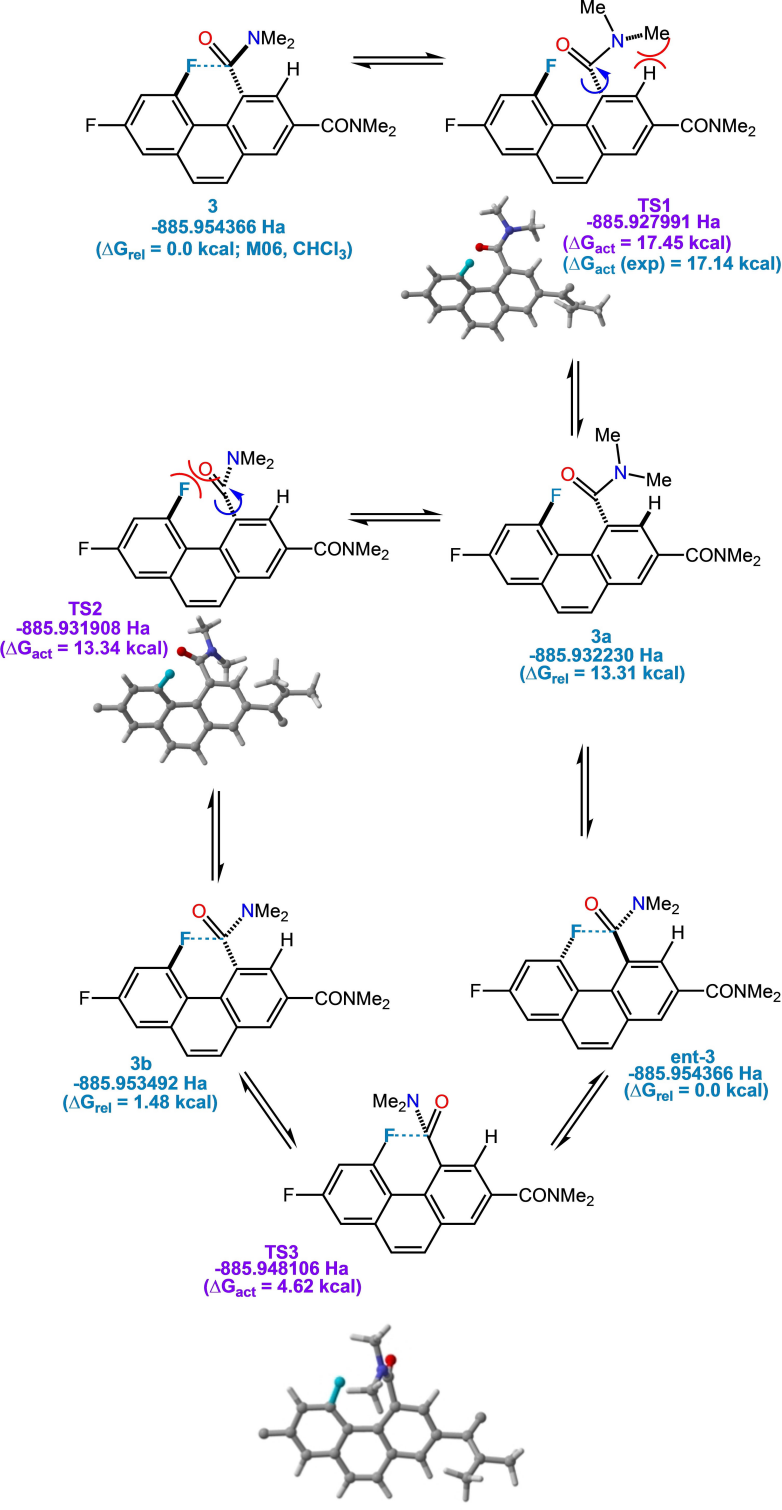
Postulated mechanism of racemization of **3** (calcs. performed at M062X/6‐311++G**).

**Figure 10:**
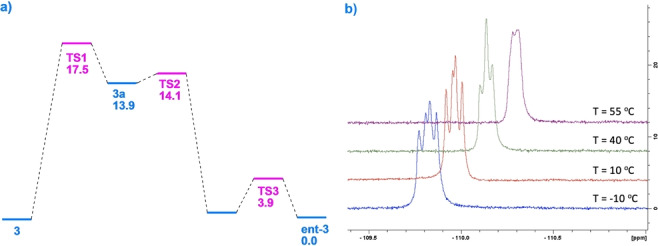
a) Calculated energy diagram for racemization of **3** (M062X/6‐311++G**). b) VT ^19^F NMR of **3**’s enantiomerization process.

Having a small, well‐defined structure possessing a correspondingly strong interaction provides an opportunity for highly illuminating gas phase ion studies (Figure 11). We subjected **3** to electrospray ionization; calculations predict protonation at the perturbed amide group favored over the distal by 2.74 kcal mol^−1^ (M06) resulting in a molecular ion **9** (357.13979 m/e). Fragment ions are interesting species in their own right, being potentially stabilized by the C−F bond. This is demonstrated by the relatively facile fragmentation to C−F bond stabilized acylium **10** which occurs at (312.08205 m/e), followed by fragmentation to C−F stabilized aryl cation **11** (284.08727 m/e). Although similar fragmentations occur in control diamide **8**, they are much less intense under identical ionization conditions.

**Figure 11:**
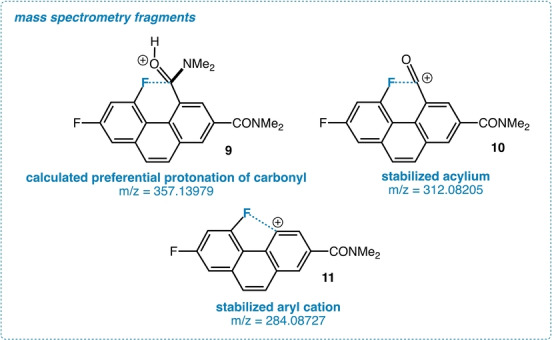
Gas phase ions.

In conclusion, we presented the first example of a close, well‐defined C−F—amide interaction that allowed both crystallographic and spectroscopic characterization, as well as facilitating an investigation of its dynamic NMR properties and reaction chemistry. This report complements extant computational studies^[4]^ and crystallographic surveys[^6^, ^10^] by providing an intimate view of this elusive yet significant contact. Given the relevance of C−F‐functional group interactions, this study should lead to a greater understanding of the unique effects of fluorine in organic and medicinal chemistry. Further studies will seek to explore additional aspects of reactivity and spectroscopy.

## Supporting Information

The Supporting Information containing experimental procedures, spectra, and computational data at https://doi.org/10.1002/anie.202207966.

## Conflict of interest

The authors declare no competing financial interest.

## Supporting information

As a service to our authors and readers, this journal provides supporting information supplied by the authors. Such materials are peer reviewed and may be re‐organized for online delivery, but are not copy‐edited or typeset. Technical support issues arising from supporting information (other than missing files) should be addressed to the authors.

Supporting Information

## Data Availability

The data that support the findings of this study are available in the Supporting Information of this article.
