# Supplementary material for: The Close Interaction of a C−F Bond with an Amide Carbonyl: Crystallographic and Spectroscopic Characterization
Source: Angew Chem Int Ed Engl. 2022 Jul 13;61(34):e202207966. doi: 10.1002/anie.202207966 (PMC9544880; doi:10.1002/anie.202207966)
Supplement: Supplementary file 1 — Supporting Information [file ANIE-61-0-s001.pdf]

Angewandte Chemie
Eine Zeitschrift der Gesellschaft Deutscher Chemiker

Supporting Information

The Close Interaction of a C–F Bond with an Amide Carbonyl: Crystallographic and Spectroscopic Characterization

*S. A. Harry, M. Kazim, P. M. Nguyen, A. Zhu, M. R. Xiang, J. Catazaro, M. Siegler, T. Lectka**

Supporting Information

Contents

General Methods 2

Single Crystal X-Ray Crystallography 2

Experimental Section 9

NMR Spectra 12

UV-Vis and IR Spectra 24

HOESY Spectra 25

Lewis Acid (BBr_3) Experiment NMR 24

Amide Isomerization & Enantiomerization 29

Computational Work 32

Coordinates of Calculated Structures 34

General Methods

Unless otherwise stated, all reactions were carried out under strictly anhydrous, air-free conditions under nitrogen. All solvents and reagents were dried and degassed by standard methods. ^{1}H and ^{13}C spectra were acquired on a 400 MHz NMR whereas ^{19}F NMR spectra were acquired on a 300 MHz NMR in $CDCl_3$ or CD_3CN at 25 °C (unless otherwise stated). The ^{1}H, ^{13}C and ^{19}F chemical shifts are given in parts per million (δ) with respect to an internal tetramethylsilane (TMS, δ 0.00 ppm) standard. NMR data are reported in the following format: chemical shifts (multiplicity (s = singlet, d = doublet, t = triplet, q = quartet m = multiplet), integration, coupling constants [Hz]). IR data were obtained using an FT-IR with a flat CaF_2 cell. HRMS data were obtained on a Thermo Scientific Q-Exactive Orbitrap mass spectrometer. All measurements were recorded at 25 °C unless otherwise stated. Spectral data were processed with ACD/NMR Processor Academic Edition.

Single Crystal X-Ray Crystallography

Crystal Structure of Compound 3.

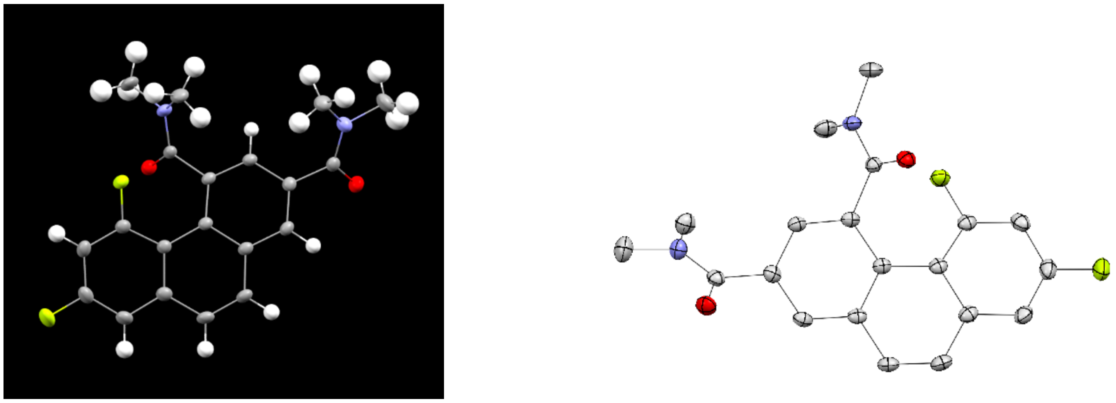

***Figure S1**: Crystal Structure of Diamide Di F and its displacement ellipsoid (50% probability level) (Compound **3**)*

All reflection intensities were measured at 110 K for **3** and **3** (high resolution, θ_{max} = 48.98°) using a SuperNova diffractometer (equipped with Atlas detector) with Mo *K*α radiation (λ = 0.71073 Å) under the program CrysAlisPro (Version CrysAlisPro 1.171.39.29c, Rigaku OD, 2017). The same program was used to refine the cell dimensions and for data reduction. The structure was solved with the program SHELXS-2018/3 (Sheldrick, 2018) and was refined on F^2 with SHELXL-2018/3 (Sheldrick, 2018). Numerical absorption correction based on gaussian integration over a multifaceted crystal model was performed using CrysAlisPro. The temperature of the data collection was controlled using the system Cryojet (manufactured by Oxford Instruments). For **3**, the H atoms were placed at calculated positions using the instructions AFIX 43 or AFIX 137 with isotropic displacement parameters having values 1.2 or 1.5 U_{eq} of the attached C atoms. The structure is ordered. For **3 (high resolution)**, the H atoms were refined freely (i.e., coordinates and isotropic temperature factor). The C–H bond distances are found within the range 0.936(14)–1.005(14) Å. The structures of **3** and **3 (high resolution)** are ordered.

*MoPro refinements for **3 (high resolution)*** - A multipolar refinement was performed using the refined ShelXL model as a starting point. The charge density was refined against structure factors using the program MoPro,[1] which experimentally determined the deformation electron density maps based on a non-spherical model of the atomic electron density.[2] The multipolar Hansen-Coppens model describes the crystal electron density as a superposition of the aspherical pseudo-atoms each modelled on a multipole expansion:

$$\rho_{atom}(r) = \rho_{core}(r) + P_{val}\kappa^3\rho_{val}(\kappa r) + \Sigma_l \kappa'^3 R_1(\kappa' r)\Sigma_m P_{lm\pm} Y_{lm\pm}(\theta, \varphi)$$

where the first two terms are the spherically averaged Hartree-Fock core and valence electron densities of the atom, and the last term is concerned with the non-spherical valence density, which is modelled using spherical harmonics functions $Y_{lm\pm}$. P_{val} is the valence population, $P_{lm\pm}$'s are the multipole populations, κ and κ' are contraction/expansion parameters, and R_1 are the radial Slater-type functions.

Before carrying out the multipolar refinement, the values of the U^{ij} parameters for H atoms were constrained to the values obtained from the SHADE server.[3] All C–H distances were restrained to fit neutron diffractions studies with a restraint sigma of 0.002 Å.[4] Those sets of constraints and restraints were kept until the end of the refinement.

The multipolar refinement was carried out using the octupole level (l = 3) for non-H atoms (C, N, O and F), and the dipole level (l = 1) for H atoms. The core and valence spherical scattering factors were calculated from the Su & Coppens.[5] All parameters (scale factor, XYZ, U^{ij}, P_{val}, P_{lm}, κ and κ') were refined successfully until convergence was reached. For the H atoms, the κ *and* κ' values were restrained to 1.16(3).[6] **Table S3** includes the crystallographic statistics obtained from the multipolar refinement.

---

[1] C. Jelsch, B. Guillot, A. Lagoutte, C. Lecomte, J. Appl. Cryst. 2005, 38, 38-54.
[2] N. K. Hansen, P. Coppens, Acta Cryst. 1978, A34, 909-921.
[3] A. Ø. Madsen, J. Appl. Cryst. 2006, 39, 757-758.
[4] F. H. Allen, Acta Cryst. 1986, B42, 515-522.
[5] Z. Su, P. Coppens, Acta Cryst. 1998, A54, 646-652.
[6] R. F. Stewart, Acta Cryst. 1976, A32, 565-574.

Table S1. Crystallographic data for 3 (ShelXL refinement)

	**3**
Crystal data	
Chemical formula	$C_{20}H_{18}F_2N_2O_2$
M_r	356.36
Crystal system, space group	Monoclinic, $P2_1/n$
Temperature (K)	110
a, *b*, *c* (Å)	11.2164 (3), 11.7704 (3), 13.3440 (3)
β (°)	97.951 (2)
V (Å^3)	1744.76 (8)
Z	4
Radiation type	Mo *K*α
μ (mm^{-1})	0.10
Crystal size (mm)	0.30 × 0.25 × 0.20
Data collection	
Diffractometer	SuperNova, Dual, Cu at zero, Atlas
Absorption correction	Gaussian<br>*CrysAlis PRO* 1.171.39.46 (Rigaku Oxford Diffraction, 2018) Numerical absorption correction based on gaussian integration over a multifaceted crystal model Empirical absorption correction using spherical harmonics, implemented in SCALE3 ABSPACK scaling algorithm.
T_{min}, T_{max}	0.541, 1.000
No. of measured, independent and observed [$I > 2\sigma(I)$] reflections	24993, 3994, 3424
R_{int}	0.029
$(\sin \theta/\lambda)_{max}$ (Å^{-1})	0.650
Refinement	
$R[F^2 > 2\sigma(F^2)]$, $wR(F^2)$, S	0.040, 0.112, 1.05
No. of reflections	3994
No. of parameters	239
H-atom treatment	H-atom parameters constrained
$\Delta\rho_{max}$, $\Delta\rho_{min}$ (e Å^{-3})	0.33, -0.20

Table S2. Crystallographic data for 3 (high resolution) (ShelXL refinement)

	**3 (high resolution)**
Crystal data	
Chemical formula	$C_{20}H_{18}F_2N_2O_2$
M_r	356.36
Crystal system, space group	Monoclinic, $P2_1/n$
Temperature (K)	110
a, *b*, *c* (Å)	11.2010 (2), 11.7537 (2), 13.3352 (2)
β (°)	97.9274 (16)
V (Å^3)	1738.84 (5)
Z	4
Radiation type	Mo *K*α
μ (mm^{-1})	0.10
Crystal size (mm)	0.53 × 0.45 × 0.31
Data collection	
Diffractometer	SuperNova, Dual, Cu at zero, Atlas
Absorption correction	Gaussian<br>*CrysAlis PRO* 1.171.41.93a (Rigaku Oxford Diffraction, 2020) Numerical absorption correction based on gaussian integration over a multifaceted crystal model Empirical absorption correction using spherical harmonics, implemented in SCALE3 ABSPACK scaling algorithm.
T_{min}, T_{max}	0.200, 1.000
No. of measured, independent and observed [$I > 2\sigma(I)$] reflections	95342, 17430, 12063
R_{int}	0.041
$(\sin \theta/\lambda)_{max}$ (Å^{-1})	1.062
Refinement	
$R[F^2 > 2\sigma(F^2)]$, $wR(F^2)$, S	0.050, 0.151, 1.05
No. of reflections	17430
No. of parameters	307
H-atom treatment	All H-atom parameters refined
$\Delta\rho_{max}$, $\Delta\rho_{min}$ (e Å^{-3})	0.67, -0.25

Table S3. Crystallographic statistics for 3 (high resolution) from the MoPro refinement

	**3 (high resolution)**
Refinement	
No. of unique reflections effectively used	16158
No. of parameters	847
$R[F^2 > 2\sigma(F^2)]$, $wR(F^2)$, S	0.037, 0.045, 1.02
H-atom treatment	All H-atom parameters refined
$\Delta\rho_{max}$, $\Delta\rho_{min}$ (e Å^{-3}), rms	0.391, -0.321, 0.072

Crystal Structure of Compound 4

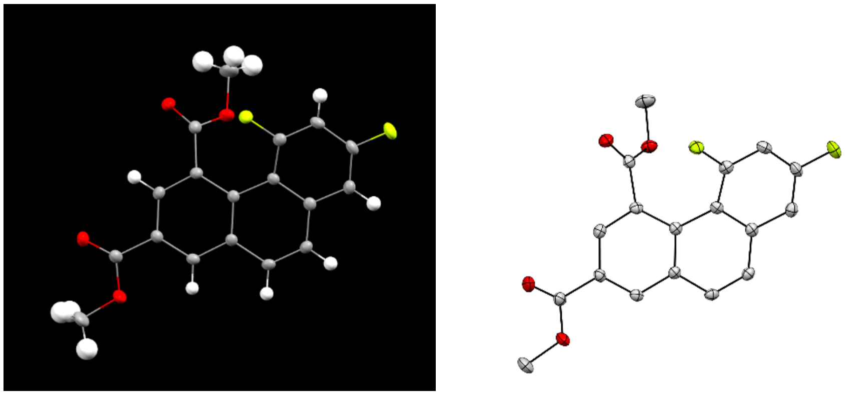

***Figure S2**: Crystal structure of Diester di F and its displacement ellipsoid (50% probability level) (Compound **4**)*

All reflection intensities were measured at 110(2) K for **4** using a SuperNova diffractometer (equipped with Atlas detector) with Mo *K*α radiation (λ = 0.71073 Å) under the program CrysAlisPro (Version CrysAlisPro 1.171.39.29c, Rigaku OD, 2017). The same program was used to refine the cell dimensions and for data reduction. The structure was solved with the program SHELXS-2018/3 (Sheldrick, 2018) and was refined on F^2 with SHELXL-2018/3 (Sheldrick, 2018). Numerical absorption correction based on gaussian integration over a multifaceted crystal model was applied using CrysAlisPro. The temperature of the data collection was controlled using the system Cryojet (manufactured by Oxford Instruments). The H atoms were placed at calculated positions using the instructions AFIX 43 or AFX 137 with isotropic displacement parameters having values 1.2 or 1.5 U_{eq} of the attached C atoms. The structure is ordered.

Table S4. Crystallographic data for 4 (ShelXL refinement)

	**4**
Crystal data	
Chemical formula	$C_{18}H_{12}F_2O_4$
M_r	330.28
Crystal system, space group	Monoclinic, $P2_1/c$
Temperature (K)	110
a, *b*, *c* (Å)	9.9816 (6), 7.0631 (3), 20.6277 (11)
β (°)	91.369 (5)
V (Å^3)	1453.86 (13)
Z	4
Radiation type	Mo *K*α
μ (mm^{-1})	0.12

Crystal size (mm)	0.29 × 0.19 × 0.04
Data collection	
Diffractometer	SuperNova, Dual, Cu at zero, Atlas
Absorption correction	Gaussian<br>*CrysAlis PRO* 1.171.41.93a (Rigaku Oxford Diffraction, 2020) Numerical absorption correction based on gaussian integration over a multifaceted crystal model Empirical absorption correction using spherical harmonics, implemented in SCALE3 ABSPACK scaling algorithm.
T_{min}, T_{max}	0.586, 1.000
No. of measured, independent and observed [$I > 2\sigma(I)$] reflections	17281, 3336, 2617
R_{int}	0.041
$(\sin \theta/\lambda)_{max}$ (Å^{-1})	0.650
Refinement	
$R[F^2 > 2\sigma(F^2)]$, $wR(F^2)$, S	0.044, 0.122, 1.04
No. of reflections	3336
No. of parameters	219
H-atom treatment	H-atom parameters constrained
$\Delta\rho_{max}$, $\Delta\rho_{min}$ (e Å^{-3})	0.34, -0.24

Computer programs: *CrysAlis PRO* 1.171.39.29c (Rigaku OD, 2017), *SHELXS2018*/3 (Sheldrick, 2018), *SHELXL2018*/3 (Sheldrick, 2018), *SHELXTL* v6.10 (Sheldrick, 2008).

Experimental Section

Synthesis and Characterization of Compounds

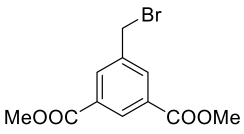

Dimethyl-5-(bromomethyl)isophthalate (Compound A): Compound **A** was synthesized from isphthalic acid following previously reported protocols.[7]

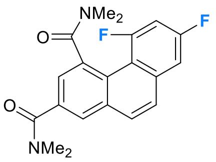

5,7-difluoro-N2,N2,N4,N4-tetramethylphenanthrene-2,4-dicarboxamide (Compound 3): Compound **A** (1g, 3.5 mmol) was dissolved in 45 mL dichloromethane containing triphenylphosphine (920 mg, 3.5 mmol) and the solution was stirred at room temperature. After the ^{1}H NMR revealed complete conversion of **A**, solvent was evaporated under reduced pressure, the mixture was dissolved in 100 mL of 1:1 MeOH and THF and lithium metal was added (74 mg, 10.5 mmol). After stirring the mixture at room temperature for 10 minutes, a solution of 3,5-difluorobenzaldehyde (500 mg, 3.5 mmol) in 15 mL THF was added. Once ^{1}H NMR revealed complete consumption of the intermediate phosphonium ylid, reaction mixture was extracted with 100 mL ethyl acetate and washed with 100 mL 1M HCl and 100 mL brine. Organic layer was dried over anhydrous sodium sulfate and evaporated under reduced pressure. This mixture was filtered through a plug of silica gel and washed with 20% ethyl acetate in hexanes to collect the cis/trans stilbene mixture which was functionalized without further purification. The mixture of cis and trans stilbene was dissolved in 60 mL water, KOH (1.2 g, 21 mmol) was added, and the mixture was refluxed for 6 hours. Thereafter, the mixture was acidified with 1M HCl, extracted with 100 mL ethyl acetate, and washed with 100 mL brine. Organic layer was dried over anhydrous sodium sulfate and evaporated under reduced pressure to obtain the intermediate cis/trans stilbene diacid. The intermediate diacid was suspended in 45 mL dichloromethane, thionyl chloride (2 mL) and DMF (4 drops) were added, and the mixture was refluxed for 4 hours. After completion, solvents were evaporated under reduced pressure, the mixture was dissolved in 200 mL cyclohexane, I_2 (100 mg) was added, and the mixture was irradiated with 254 nm light in a rayonet photochemical reactor for 14 hours. Thereafter, excess iodine was quenched with solid sodium thiosulfate, filtered, and the solvent was evaporated under reduced pressure. Finally, the mixture was dissolved in 45 mL dichloromethane, dimethylamine hydrochloride (1.7 g, 21 mmol), and sodium bicarbonate (2.5 g) were added, and the mixture was stirred at room temperature for 2 hours. The mixture was then washed with 50 mL 1M HCl, 50 mL brine, dried over anhydrous sodium sulfate and evaporated under reduced pressure. Final product (compound **3**) was purified with silica gel chromatography using ethyl acetate (60% in hexane) initially to remove by-products followed by 5% methanol in ethyl acetate as a yellow solid (105 mg, 8.5% overall yield). ^{1}H NMR ($CDCl_3$) δ 7.96 (d, J = 1.5 Hz, 1H), 7.63 – 7.78 (m, 3H), 7.36 (dd, J = 1.9 Hz, 1.2 Hz, 1H), 7.03 – 7.14 (m, 1H), 3.00 – 3.25 (m, 12H); ^{13}C NMR ($CDCl_3$) δ 172.4 (d, J = 6.3 Hz), 170.2, 162.0 (m), 159.4 (m), 136.2 (m), 135.8 (m), 133.8, 133.3, 129.2, 128.5, 127.3 (m), 126.1, 125.3, 115.2 (m), 109.0 (m), 103.4 (m), 39.8 (m), 39.7, 35.5, 35.0; ^{19}F NMR ($CDCl_3$) δ –103.7 (1F, m), –110.1 (1F, m); ^{19}F NMR {^{1}H} ($CDCl_3$) δ –103.7 (1F, d), –110.1 (1F, d); IR 3064, 2826, 1622, 1603 (cm^{-1}, CaF_2, CH_2Cl_2); FTMS + p (ESI) calc for $C_{20}H_{19}O_2N_2F_2^+$: 357.1415, found 357.1407.

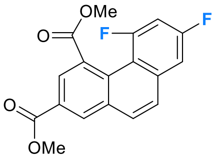

[7] Feng, Y.; Liu, Z.; Chen, H.; Yan, Z.; He, Y.; Liu, C.; Fan, Q. *Chem. Eur. J.* **2014**, *20*, 7069-7082.

Dimethyl 5,7-difluorophenanthrene-2,4-dicarboxylate (Compound 4): Compound **4** was synthesized following similar protocol to the synthesis of compound **3** with a modification of the final step. After cyclization in the rayonet photochemical reactor, the mixture was dissolved in 45 mL methanol and the mixture was stirred at room temperature for 2 hours. Solvent was then evaporated under reduced pressure, reaction mixture was extracted with 45 mL ethyl acetate and washed with 45 mL 1M HCl, 45 mL brine, dried over anhydrous sodium sulfate and evaporated under reduced pressure. Product **4** was purified using silica gel chromatography with 10% ethyl acetate in hexanes as a light-yellow solid. ^{1}H NMR ($CDCl_3$) δ 8.62 – 8.66 (m, 1H), 8.60 (d, J = 1.7 Hz, 1H), 7.67 – 7.86 (m, 2H), 7.39 (dd, J = 2 Hz, 8.5 Hz, 1H), 7.05 – 7.16 (m, 1H), 4.01 (s, 3H), 3.94 (s, 3H); ^{13}C NMR ($CDCl_3$) δ 169.1 (d, J = 3.7 Hz), 165.9, 162.5 (m), 160.0 (m), 164.4 (m), 133.1, 132.5, 132.0 (d), 129.0, 128.96, 127.7, 127.3 (m), 127.27 (m), 52.5, 52.4; ^{19}F NMR ($CDCl_3$) δ –101.6 (1F, m), –108.5 (1F, m); ^{19}F NMR {^{1}H} ($CDCl_3$) δ –101.6 (1F, d), –108.5 (1F, d); FTMS + p (ESI) calc for $C_{18}H_{13}O_4F_2^{+}$: 331.0782, found 331.0779.

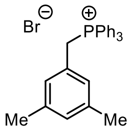

(3,5-dimethylbenzyl)triphenylphosphonium bromide (Compound B): Compound **B** was synthesized following previously reported protocols.[8]

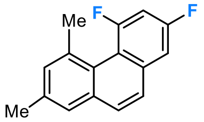

2,4-difluoro-5,7-dimethylphenanthrene (Compound C): Compound **B** (5g, 10.84 mmol) was dissolved in 100 mL (1:1) mixture of THF and methanol and lithium metal (230 mg, 32.5 mmol) were added to the mixture. After stirring the mixture at room temperature for 10 minutes, a solution of 3,5-difluorobenzaldehyde (1.54g, 10.84 mmol) was added dropwise and the mixture was stirred at room temperature overnight. The mixture was then extracted with 100 mL ethyl acetate and washed with 100 mL 1M HCl, 100 mL brine, dried over anhydrous sodium sulfate and evaporated under reduced pressure. The mixture was then filtered over a plug of silica gel with hexanes to collect the cis and trans dimethyl stilbene intermediate. After complete evaporation of the solvents, the cis/trans dimethyl stilbene was dissolved in 400 mL cyclohexanes, I_2 (80 mg) was added, and the mixture was irradiated with 254 nm light in a rayonet photochemical reactor for 4 days. Thereafter, excess iodine was quenched with solid sodium thiosulfate and the solvents were evaporated under reduced pressure. Cyclized product Compound **C** was isolated with silica gel chromatography using hexanes as a dense colorless liquid which solidified after a few days (788 mg, 30% overall yield). Compound **C** was further purified by recrystallization in methanol at freezing temperature. ^{1}H NMR ($CDCl_3$) δ 7.59 – 7.64 (m, 1H), 7.52 (d, J = 1.9 Hz, 1H), 7.47 – 7.50 (m, 1H), 7.31 – 7.41 (m, 2H), 7.03 – 7.13 (m, 1H), 2.68 (d, J = 12.6 Hz, 3H), 2.54 (s, 3H); ^{13}C NMR ($CDCl_3$) δ 161.5 (m), 160.7 (m), 159.1 (m), 158.2 (m), 136.3, 135.5 (m), 133.1, 132.2, 129.5, 125.4, 124.9 (m), 116.1 (m), 108.0 (m), 102.6, 102.3 (m), 102.0, 23.3 (m), 21.1; ^{19}F NMR ($CDCl_3$) δ –96.6 (1F, m), –113.8 (1F, m); FTMS + p (ESI) calc for $C_{16}H_{12}F_2^{+}$: 242.2639, found 242.2844.

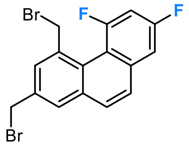

2,4-bis(bromomethyl)-5,7-difluorophenanthrene (Compound 6): Compound **C** (500 mg, 2.06 mmol) was dissolved in 50 mL anhydrous benzene containing NBS (210 mg, 4.33 mmol) and benzoyl peroxide (50 mg, 0.21 mmol) at room temperature under N_2. The mixture was then refluxed overnight under N_2. After completion, the mixture was extracted with 50 mL ethyl acetate, washed with 100 mL sodium bicarbonate solution, 100 mL brine, dried over anhydrous sodium sulfate, and the organic solvents were evaporated under reduced pressure. Dibrominated compound **6** was isolated with silica gel chromatography using 15% ethyl acetate in hexanes as a white solid (544 mg, 66% yield). ^{1}H NMR ($CDCl_3$) δ 7.99 (d, 1H, J = 1.90 Hz), 7.81 (d, 1H, J = 1.91 Hz), 7.65 – 7.70 (m, 1H), 7.58 – 7.64 (m, 1H), 7.38 – 7.43 (m,

[8] Stammel, C.; Froehlich, R.; Wolff, C.; Wenck, H.; De Meijere, A.; Mattay, J. *Eur. J. Org. Chem.*, **1999**, *7*, 1709–1718.

1H), 7.10 – 7.18 (m, 1H), 5.06 – 5.11 (d, 2H, J = 5.5 Hz), 4.72 (s, 2H); ^{13}C NMR ($CDCl_3$) δ 162.2 (m), 160.7 (m), 159.7 (m), 158.1 (m), 136.4, 132.8, 132.3, 129.1, 128.1, 126.2, 125.0, 114.9 (m), 108.8 (m), 103.2, 102.9 (m), 102.6, 34.3 (m), 32.4 (m); ^{19}F NMR ($CDCl_3$) δ –99.9 (1F, m), –110.3 (1F, m); TOF MS (FD+) calc for $C_{16}H_{10}Br_2F_2^+$: 397.9117, found 397.9102.

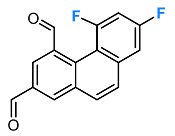

5,7-difluorophenanthrene-2,4-dicarbaldehyde (Compound 7): Compound **6** (500 mg, 1.25 mmol) was dissolved in 40 mL acetonitrile containing silver nitrate (1.1g, 6.25 mmol) at room temperature and then the mixture was refluxed for 2 hours. After the ^{1}H NMR of the mixture revealed complete conversion of compound **6**, it was filtered over celite, and the solvent was evaporated under reduced pressure. The intermediate nitrate ester was dissolved in 50 mL ethanol, Pd/C (50 mg) was added, and the mixture was treated with hydrogen gas until ^{1}H NMR revealed complete conversion to the intermediate dialcohol. After filtering out excess Pd/C and evaporation of the solvent, the mixture was dissolved in 40 mL anhydrous dichloromethane. To the mixture were added molecular sieves (2g), potassium carbonate (1.2g, 8.7 mmol) and PCC (590 mg, 2.75 mmol) and stirred at room temperature for 2 hours. Upon completion, the mixture was filtered over celite, and solvent was evaporated under reduced pressure. The final product (dialdehyde **7**) was isolated with silica gel chromatography using 10% ethyl acetate in hexanes as a light-yellow solid (277 mg, 82% yield). ^{1}H NMR ($CDCl_3$) δ 10.24–10.29 (d, J = 14 Hz, 1H), 10.27 (s, 1H), 8.49–8.63 (m, 2H), 7.88–7.96 (m, 1H), 7.78–7.86 (m, 1H), 7.45–7.53 (m, 1H), 7.15–7.24 (m, 1H); ^{13}C NMR ($CDCl_3$) δ 190.8, 189.2 (d, 15.6 Hz), 163.4 (m), 161.5 (m), 160.9 (m), 158.9 (m), 137.0 (m), 135.5 (m), 134.3, 134.1, 132.7 (m), 129.1 (m), 128.3, 127.9 (m), 114.4 (m), 109.4 (m), 104.2, 103. 9 (m), 103.6; ^{19}F NMR ($CDCl_3$) δ –105.3 (1F, m), –106.2 (1F, m); FTMS + p (ESI) calc for $C_{16}H_9O_2F_2^+$: 271.0571, found 271.0568.

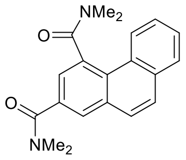

N2,N2,N4,N4-tetramethylphenanthrene-2,4-dicarboxamide (Compound 8): Compound **A** (750 mg, 2.6 mmol) was dissolved in 45 mL dichloromethane containing triphenylphosphine (690 mg, 2.6 mmol) and the solution was stirred at room temperature. After the ^{1}H NMR revealed complete conversion of **A**, solvent was evaporated under reduced pressure, the mixture was dissolved in 100 mL of (1:1) THF:MeOH and lithium metal was added (55 mg, 7.8 mmol). After stirring the mixture at room temperature for 10 minutes, a solution of benzaldehyde (0.29 mL, 2.86 mmol) in 10 mL THF was added. Once ^{1}H NMR revealed complete consumption of the intermediate phosphonium salt, reaction mixture was extracted with 100 mL ethyl acetate and washed with 100 mL 1M HCl and 100 mL brine. Organic layer was dried over anhydrous sodium sulfate and evaporated under reduced pressure. This mixture was filtered through a plug of silica gel and washed with 20% ethyl acetate in hexanes to collect the cis/trans stilbene mixture, which was dissolved in 200 mL cyclohexane, I_2 (80 mg) was added, and the mixture was irradiated with 254 nm light in a rayonet photochemical reactor for 14 hours. Thereafter, excess iodine was quenched with solid sodium thiosulfate, filtered, and the solvent was evaporated under reduced pressure. Intermediate cyclized diester was isolated with silica gel chromatography using 15% ethyl acetate in hexanes and then suspended in 40 mL water containing (230 mg, 4.1 mmol) of KOH, and the mixture was refluxed for 6 hours. After complete consumption of the diester, the mixture was acidified with 1M HCl, extracted with 50 mL ethyl acetate, washed with brine, organic layer was dried over anhydrous sodium sulfate and evaporated under reduced pressure. Intermediate diacid was suspended in 40 mL anhydrous dichloromethane, thionyl chloride (0.3 mL, 4.1 mmol) and DMF (4 drops) were added, and the mixture was refluxed under N_2 for 3 hours. After evaporating excess solvents under reduced pressure, the mixture was redissolved in 50 mL anhydrous dichloromethane, dimethylamine hydrochloride (165 mg, 2 mmol) and sodium bicarbonate (345 mg, 4.1 mmol) were added, and the mixture was stirred at room temperature for 2 hours. After completion, mixture was extracted with additional 30 mL dichloromethane, washed with 80 mL 1M HCl, 80 mL brine, dried over anhydrous sodium sulfate and evaporated under reduced pressure. Product **8** was isolated with silica gel chromatography using 10% methanol in ethyl acetate as a white solid (58 mg, 7% overall yield). ^{1}H NMR ($CDCl_3$) δ 8.38 – 8.49 (m, 1H), 7.97 – 8.03 (m, 1H), 7.87 – 7.94 (m, 1H), 7.71 – 7.83 (m, 2H), 7.52 – 7.67 (m, 3H), 3.27 (s, 3H), 3.16 (s, 3H), 3.05 (s, 3H), 2.58 (s, 3H); ^{13}C NMR ($CDCl_3$) δ 172.9, 170.5, 134.4, 134.0, 133.3, 132.8, 129.0, 128.8, 128.5, 127.4, 127.30, 127.28,

127.2, 124.9, 124.7, 39.8, 38.3, 35.5, 35.1; IR 3066, 2928, 1634 (cm^{-1}, CaF_2, CH_2Cl_2); FTMS + p (ESI) calc for $C_{20}H_{21}O_2N_2^+$: 321.1603, found 321.1600.

NMR Spectra

^{1}H Spectrum of **Compound 3** in $CHCl_3$

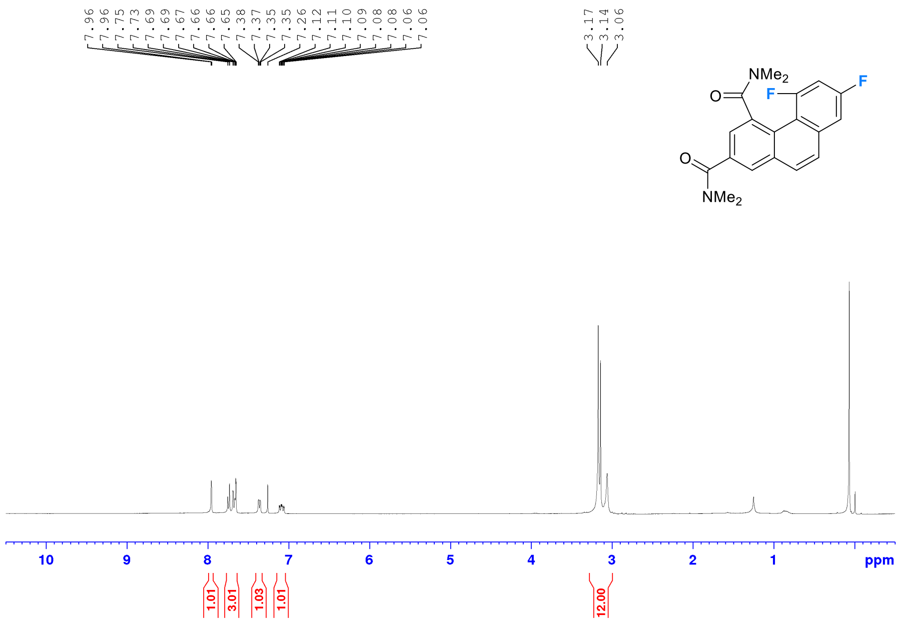

^{13}C Spectrum of **Compound 3** in $CHCl_3$

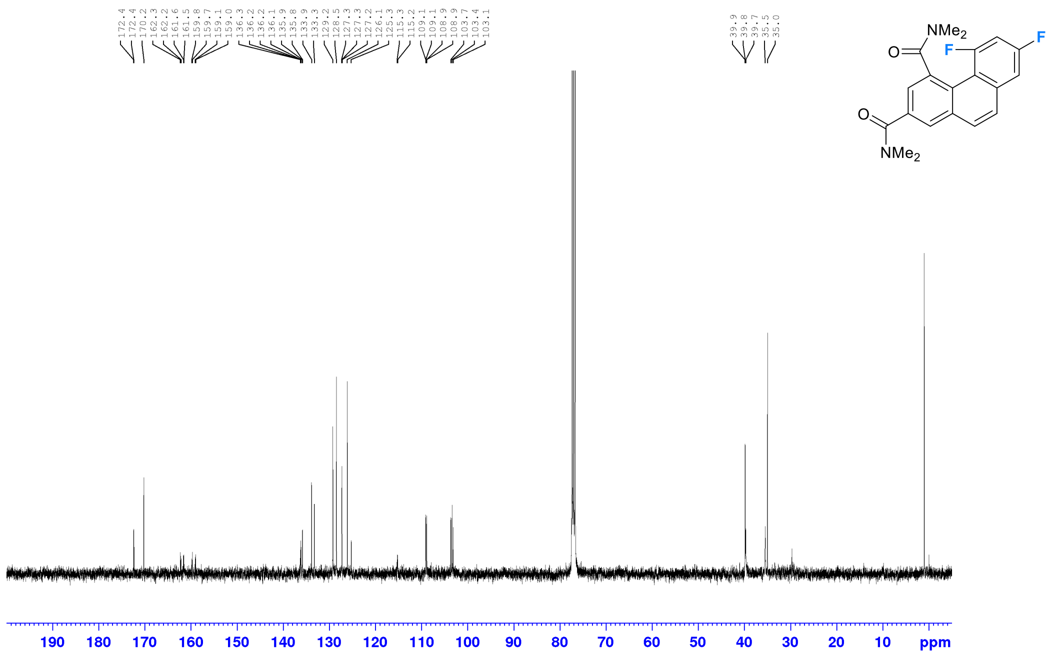
172.4
172.4
170.2
162.3
162.2
161.6
161.5
159.8
159.7
159.1
159.0
136.3
136.2
136.2
136.1
135.9
135.8
133.9
133.3
129.2
128.5
127.3
127.3
127.2
126.1
125.3
115.3
115.2
109.1
109.1
108.9
103.7
103.4
103.1
39.9
39.8
39.7
35.5
35.0
NMe2
F
F
O
O
NMe2
190
180
170
160
150
140
130
120
110
100
90
80
70
60
50
40
30
20
10
ppm

^{19}F Spectrum of **Compound 3** in $CHCl_3$

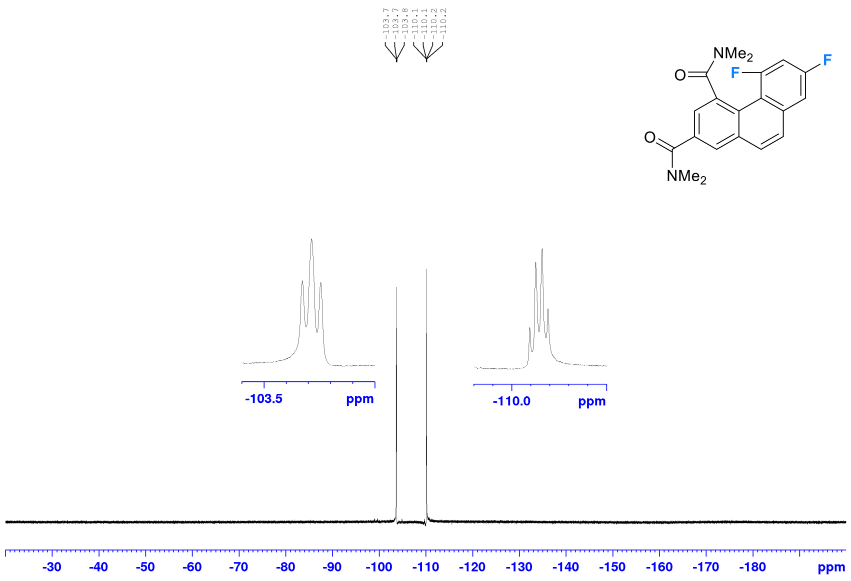

^{1}H Spectrum of **Compound 8** in $CHCl_3$

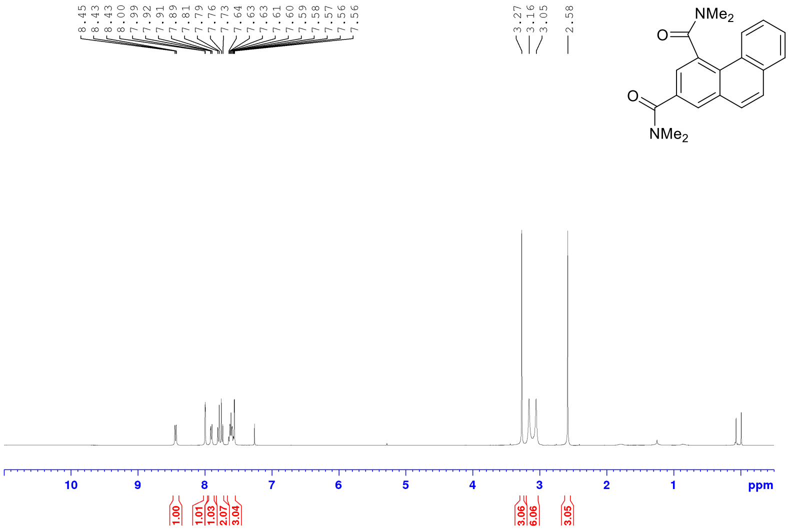

^{13}C Spectrum of **Compound 8** in $CHCl_3$

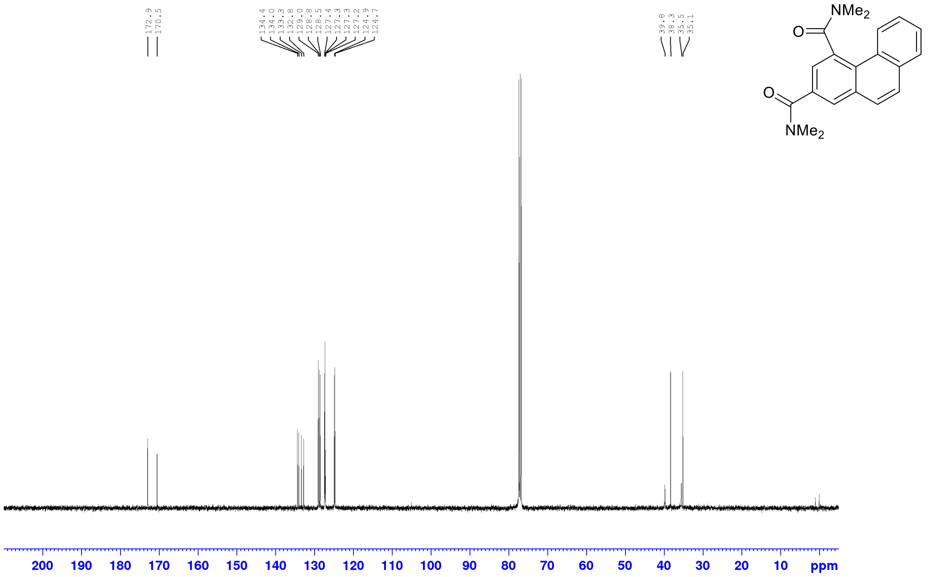

[1]H Spectrum of **Compound 4** in $CHCl_3$

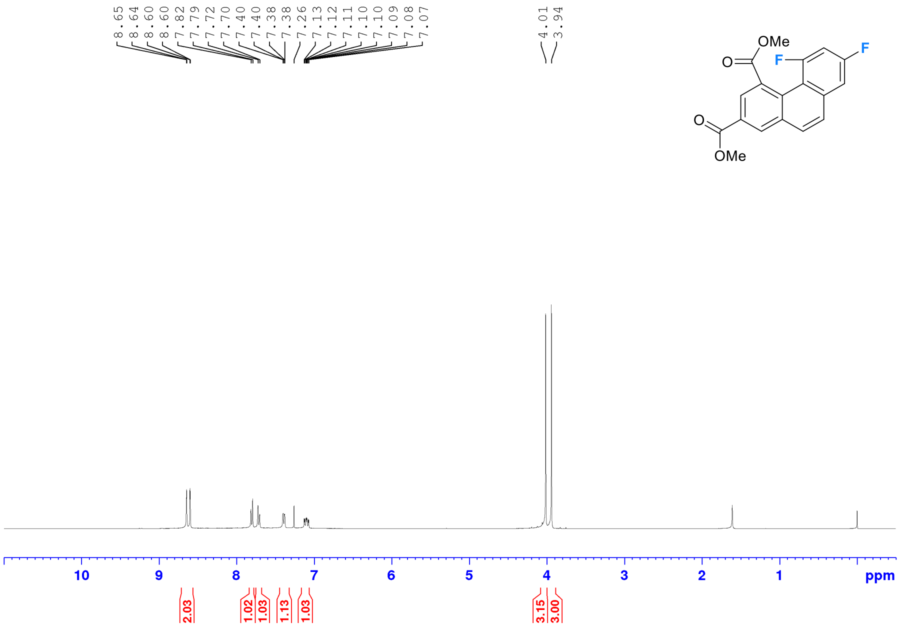

[13]C Spectrum of **Compound 4** in $CHCl_3$

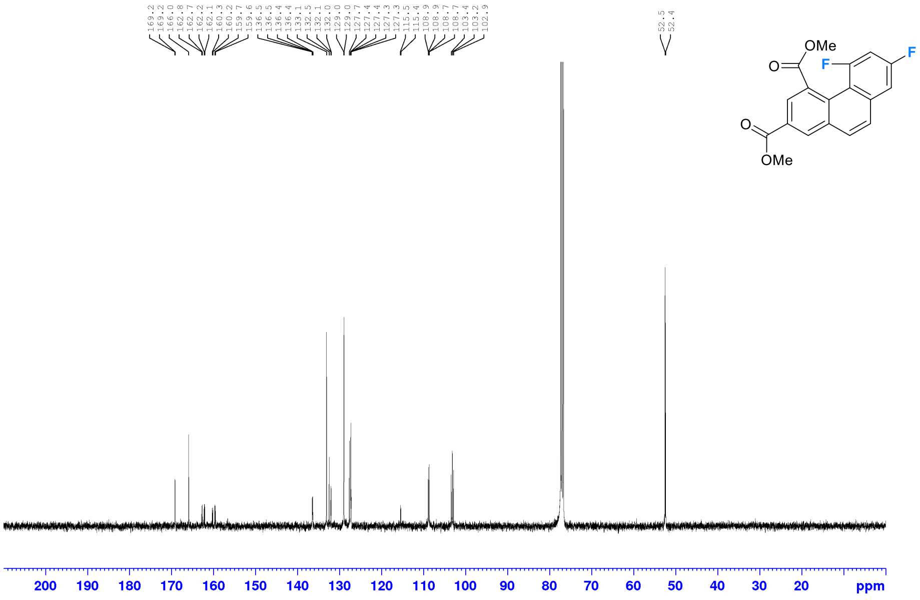

^{19}F Spectrum of **Compound 4** in $CHCl_3$

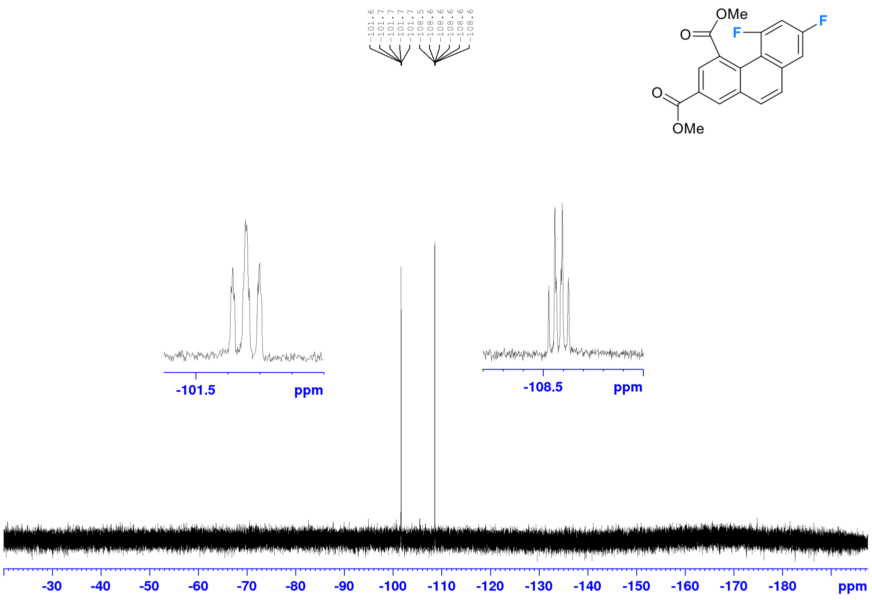

^{1}H Spectrum of **Compound C** in $CHCl_3$

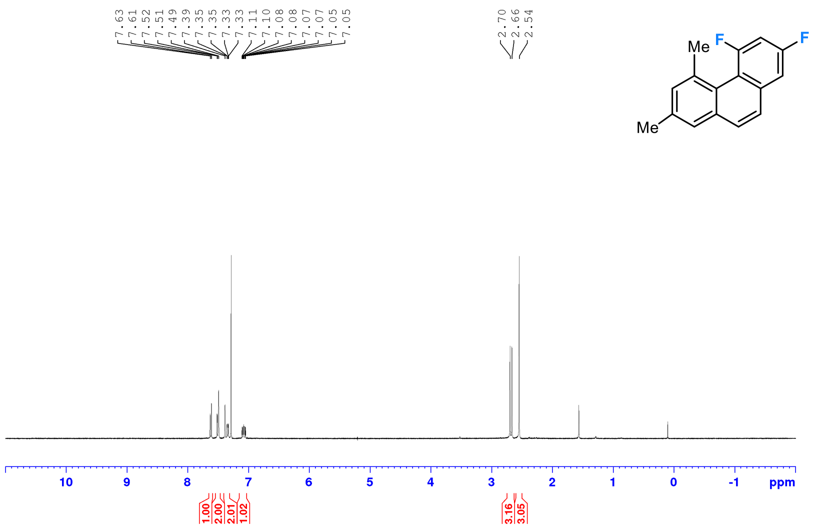

^{13}C Spectrum of **Compound C** in $CHCl_3$

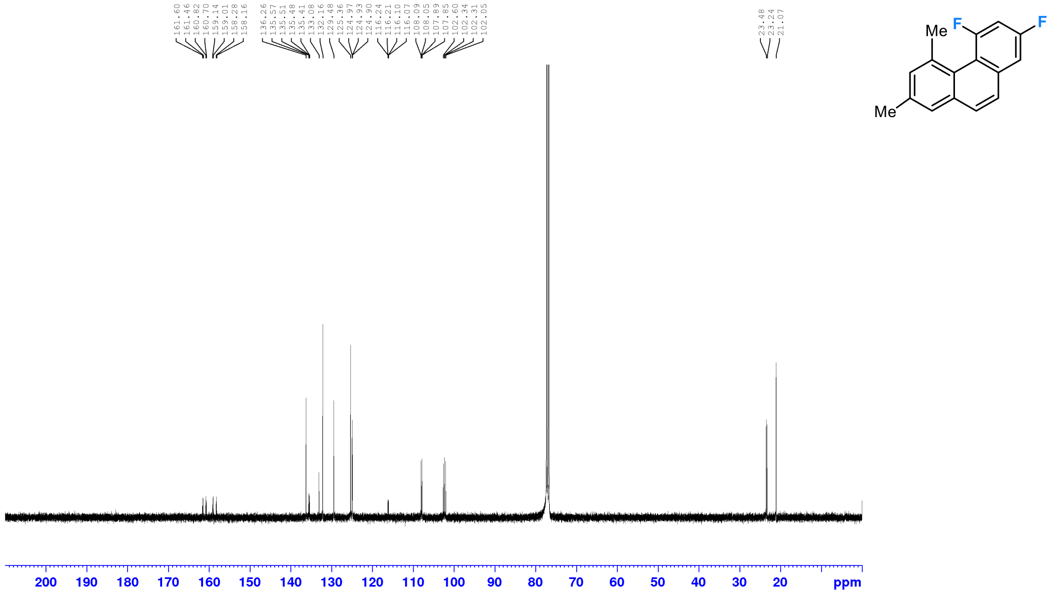

[19]F Spectrum of **Compound C** in $CHCl_3$

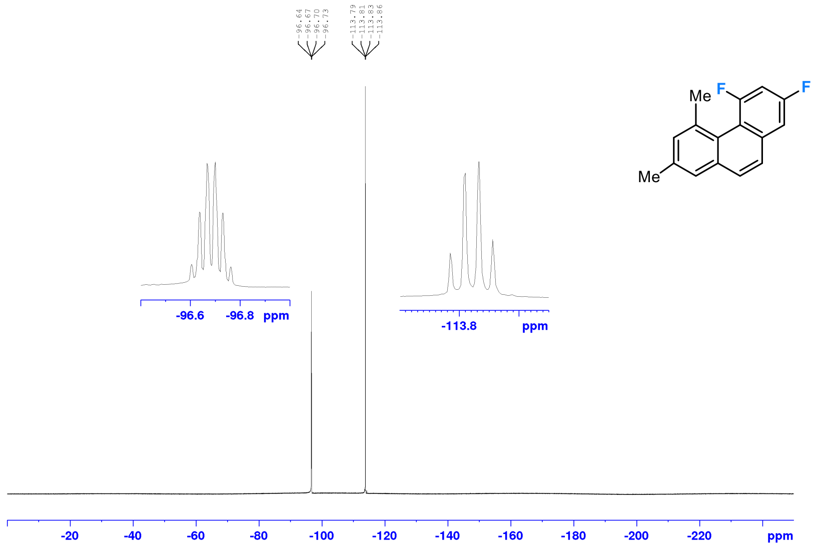

[1]H Spectrum of **Compound 6** in $CHCl_3$

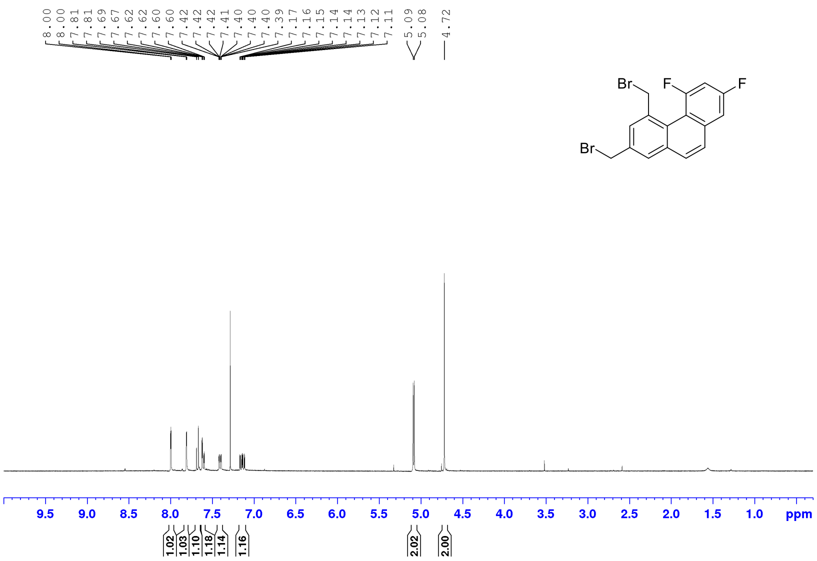

[13]C Spectrum of **Compound 6** in $CHCl_3$

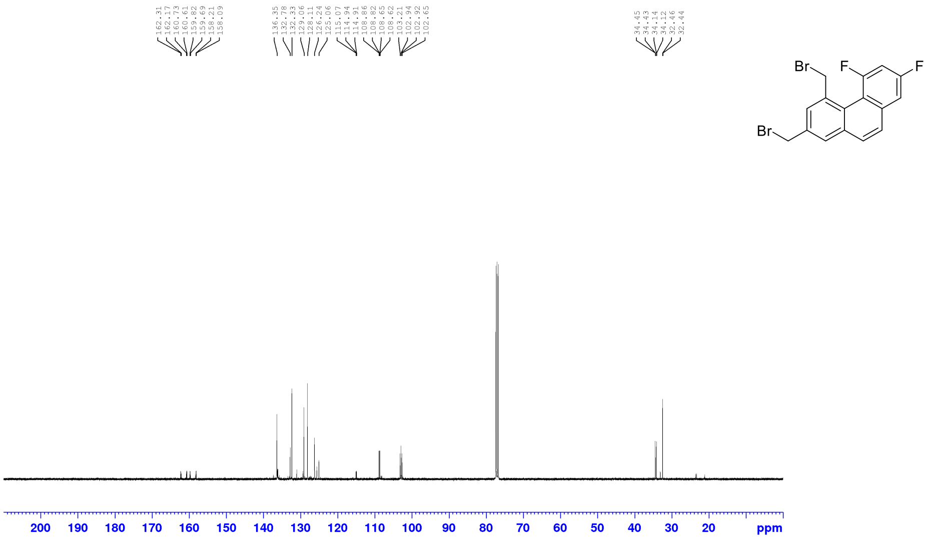

^{19}F Spectrum of **Compound 6** in $CHCl_3$

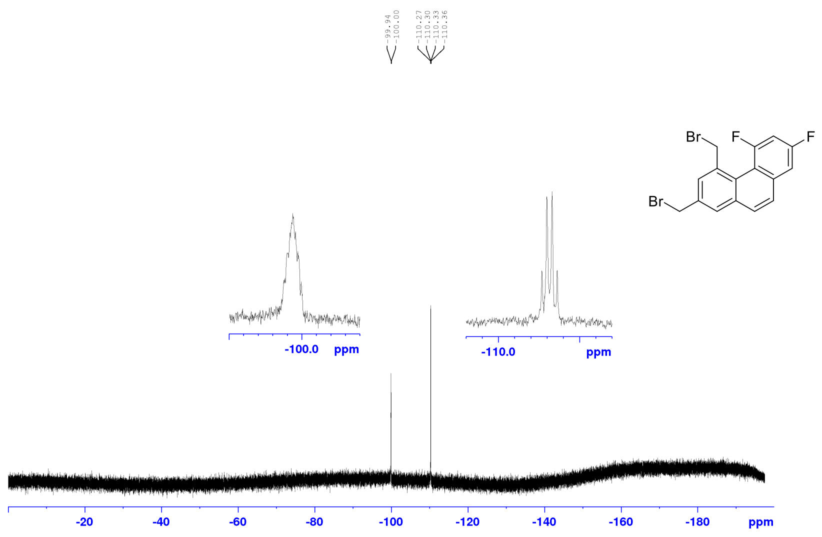

[1]H Spectrum of **Compound 7** in $CHCl_3$

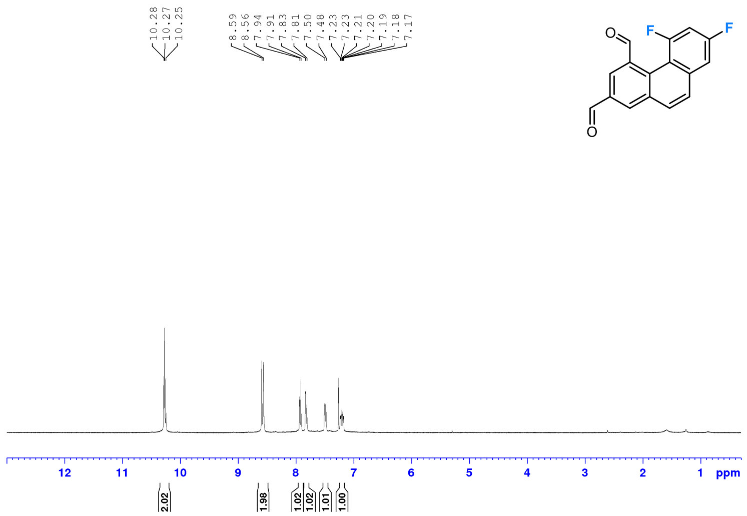

[13]C Spectrum of **Compound 7** in $CHCl_3$

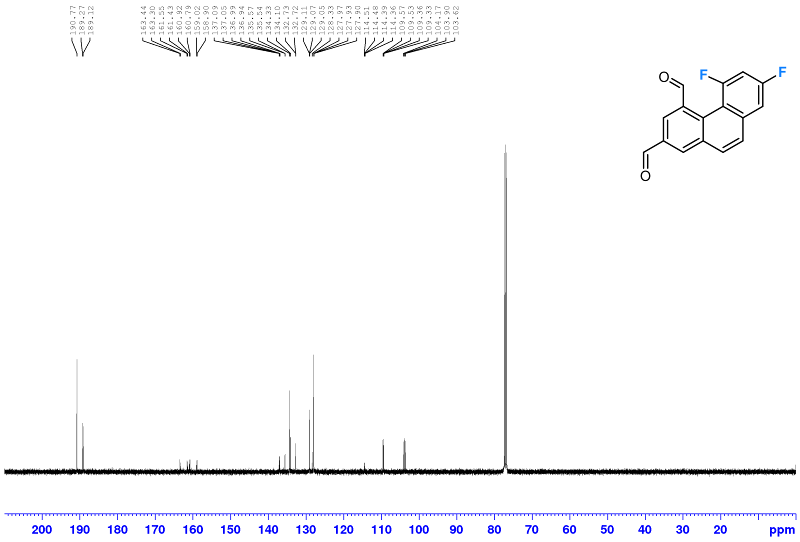

^{19}F Spectrum of **Compound 7** in $CHCl_3$

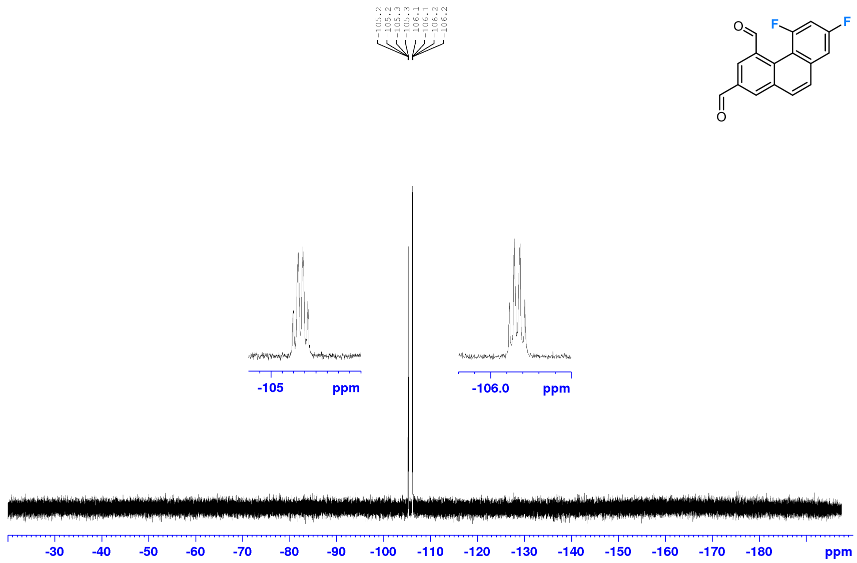

UV-Vis and IR Spectra

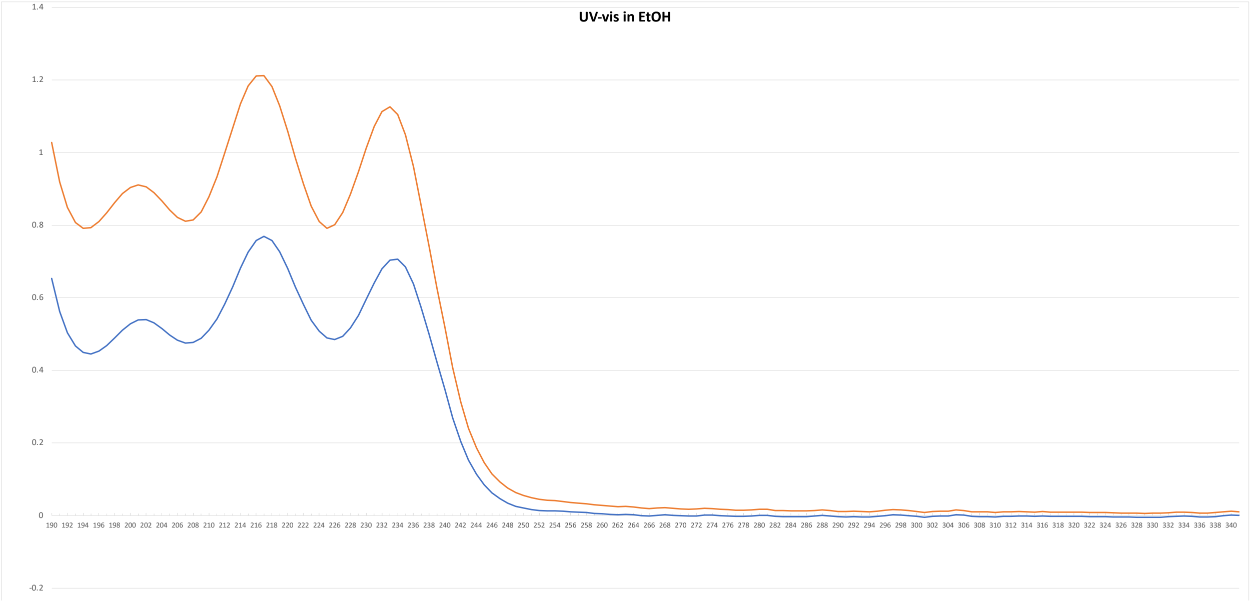

Figure S3. *UV-Vis spectra of Compound **3** (blue) and Control diamide compound **8** (orange); λ_{max} = 204, 228, 239; for **3** and λ_{max} = 204, 222, 234 for **8**.*

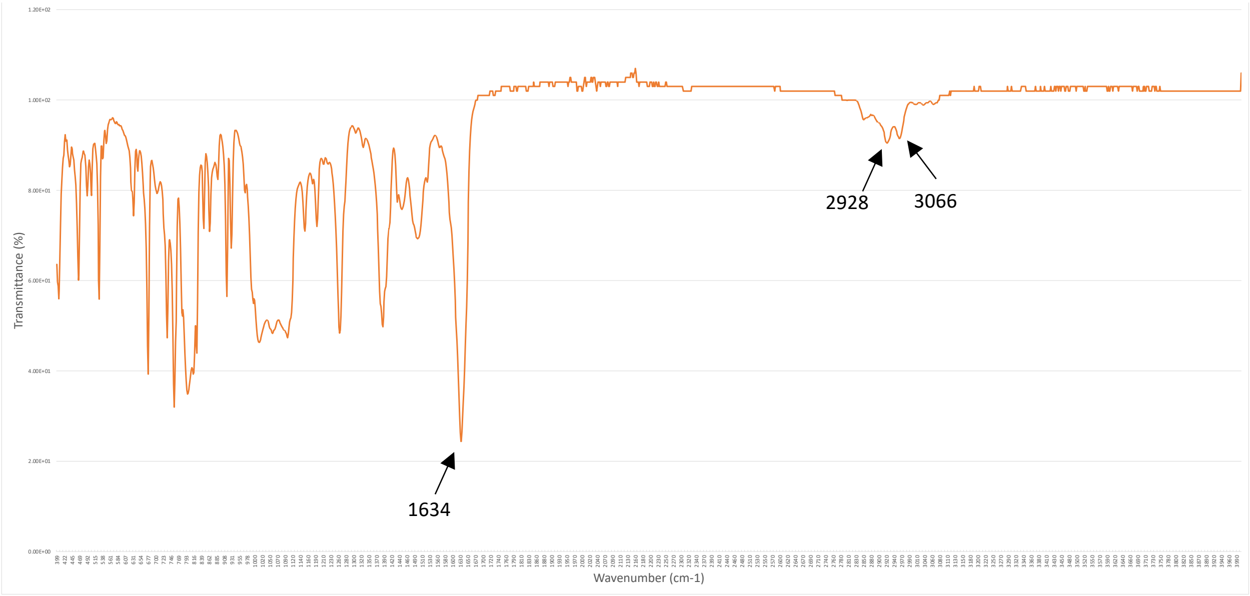

Figure S4. *FTIR spectrum of control diamide compound **8***

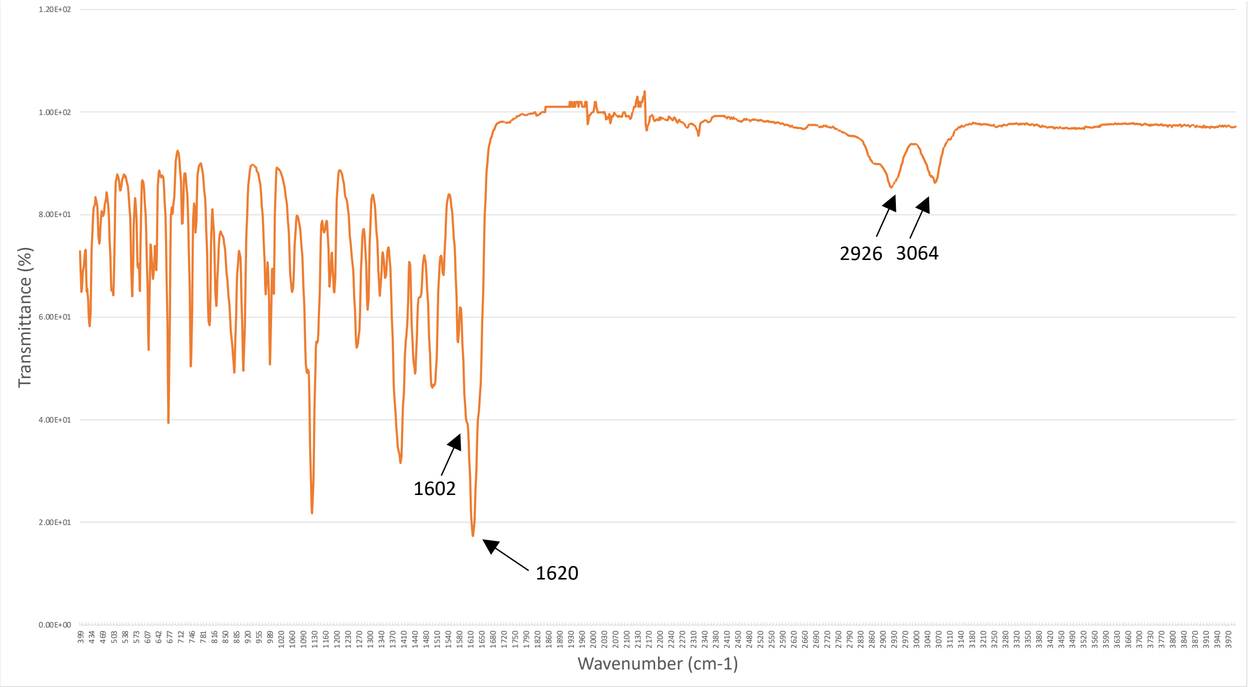

Figure S5. *FTIR spectrum of compound **3***

HOESY Spectra

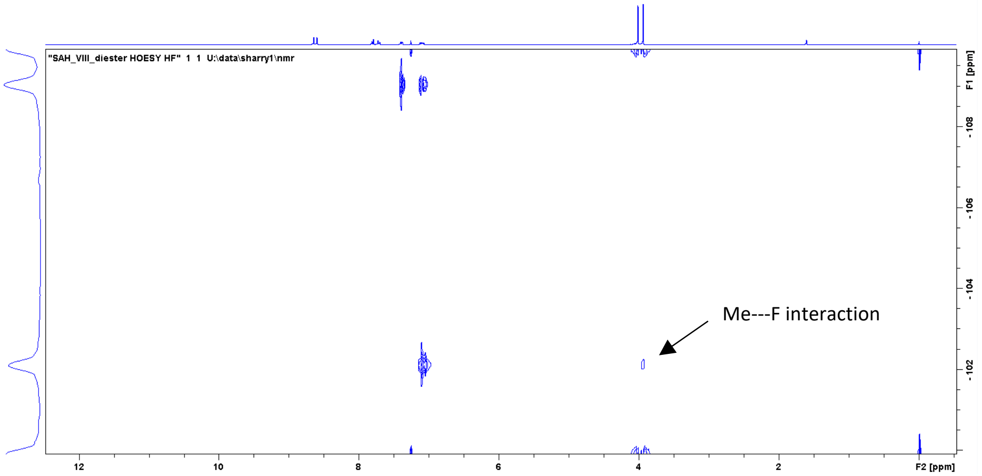

Figure S6. *HOESY spectrum of diester **4***

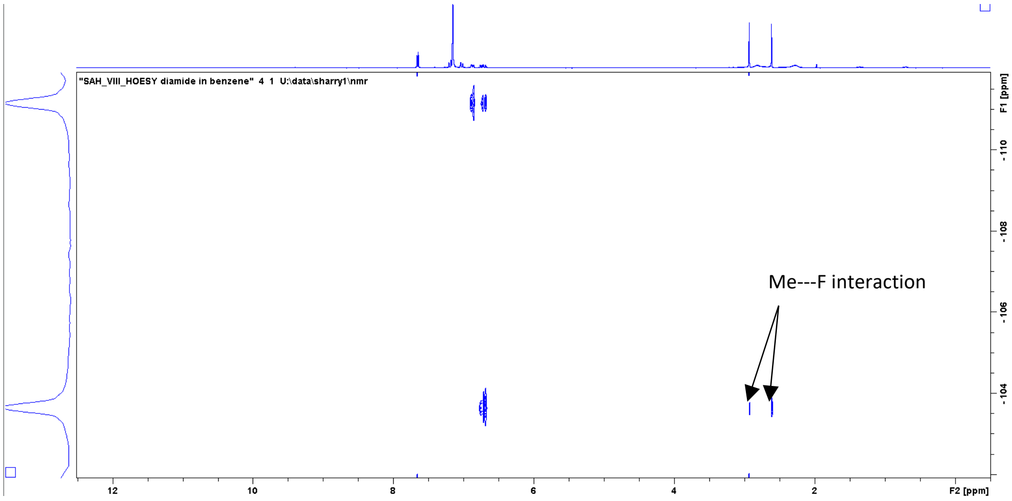

Figure S7. *HOESY spectrum of diamide* ***3***

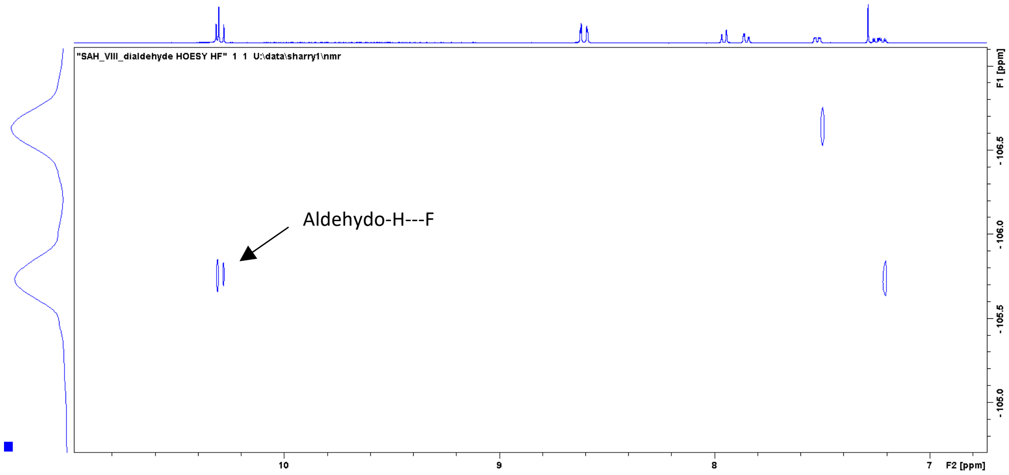

Figure S8. *HOESY spectrum of dialdehyde* ***7***

Lewis Acid (BBr_3) Experiment NMR

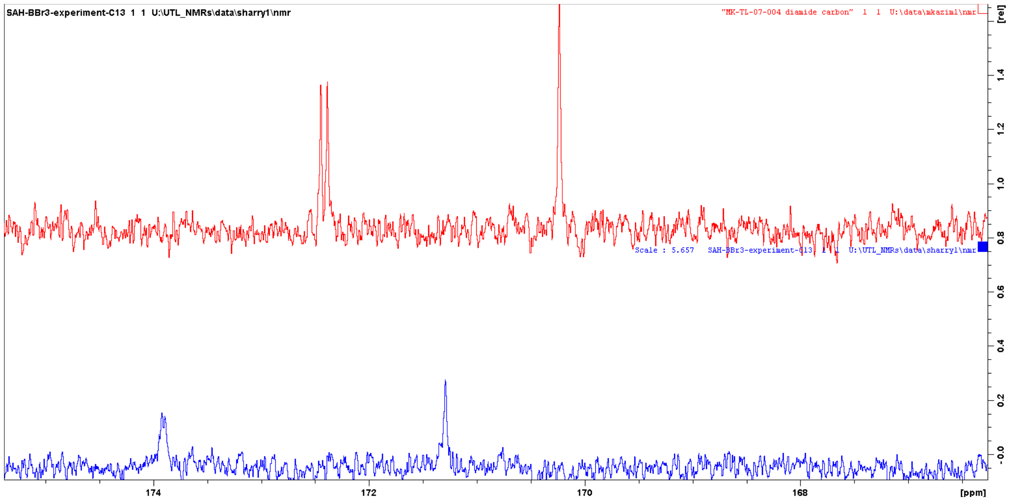

Figure S9. *Zoomed carbonyl region of free diamide compound **3** (red) and BBr_3 coordinated diamide **3–(BBr_3)$_2$** (blue) in their corresponding ^{13}C NMR spectra.*

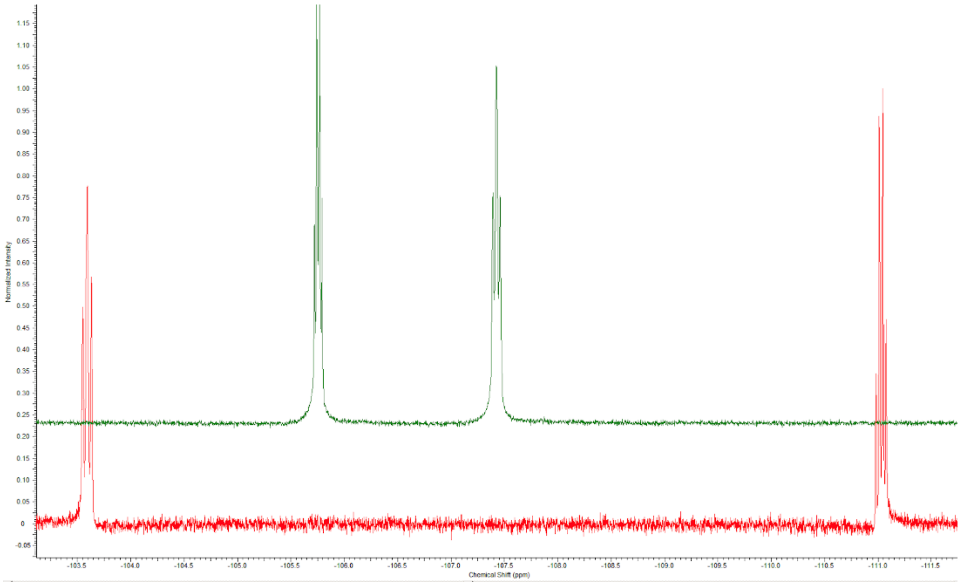

Figure S10. *^{19}F NMR spectra of free diamide **3** (red) and BBr_3 coordinated diamide **3–(BBr_3)$_2$** (green); Red: Probe F (-103.5 ppm), Control F (-111.0 ppm); Green: Probe F (-107.5 ppm), Control F (-105.7 ppm).*

Crystal Packing of the diamide 3 (Enantiomers)

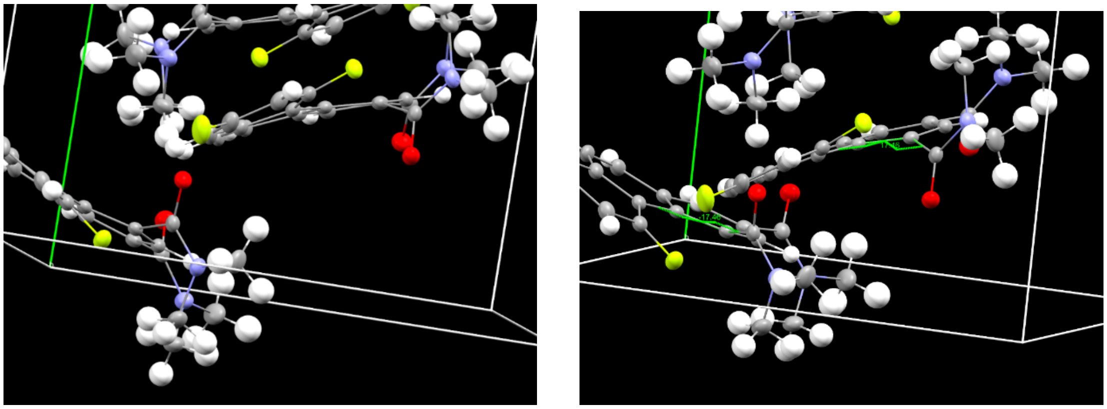

Figure S11. *Zoomed image of the diamide* ***3*** *showing two enantiomers co-crystallizing in its crystallographic unit and the aromatic distortion in those enantiomers (17.46 degrees).*

Pyramidalization of the Probe Amide in Compound 3

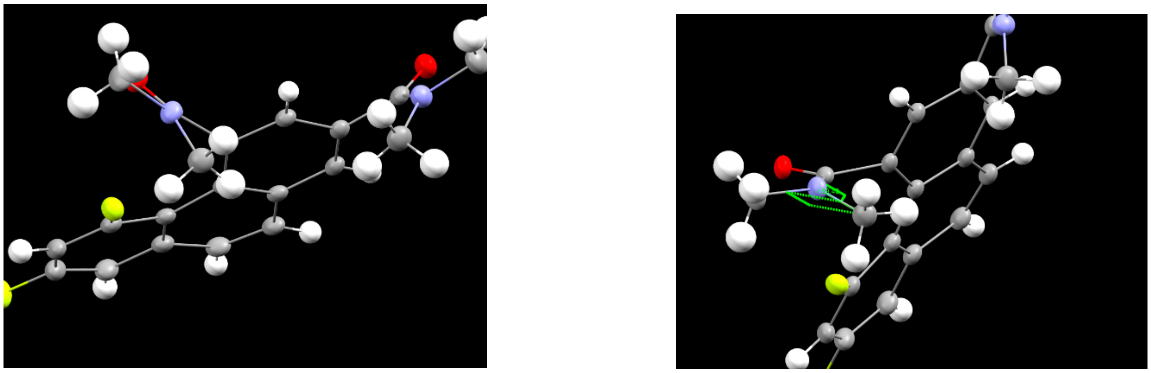

Figure S12. *Zoomed image of the probe amide in compound* ***3*** *showing its pyramidalization and the angle of distortion (159.42 degrees).*

Amide Isomerization & Enantiomerization

Line Shape Analysis for Amide Isomerization: The line shape analyses were performed using the iNMR software program (MestreLab Research, Felician Barrera 9B-Bajo 15706 Santiago de Composatela Spain).

Line Shape Analysis of 3 at 120 °C, d_6-DMSO solvent

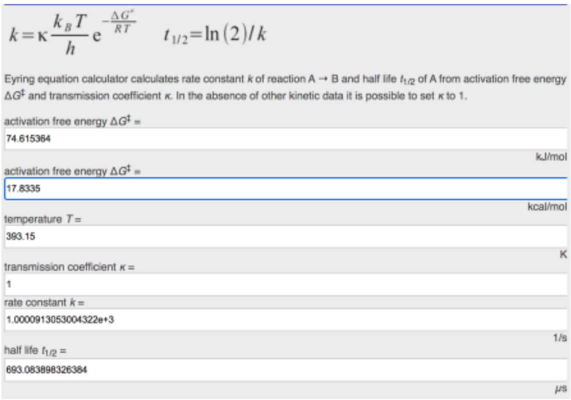

Simulated Spectrum

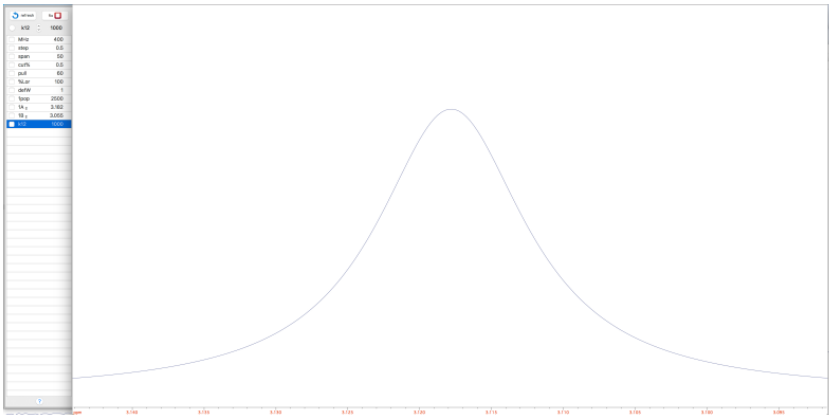

Experimental Spectrum

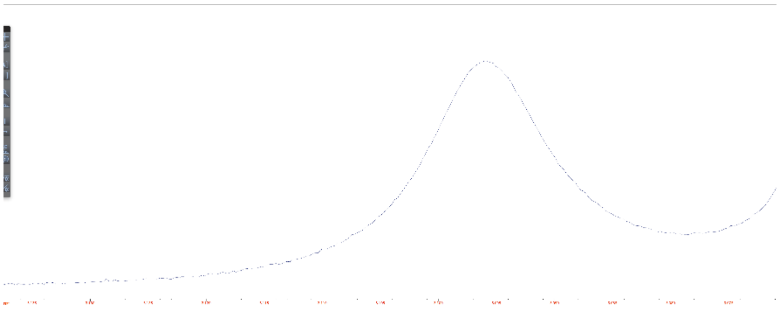

Line Shape Analysis of 8 at 120 °C, d_6-DMSO solvent

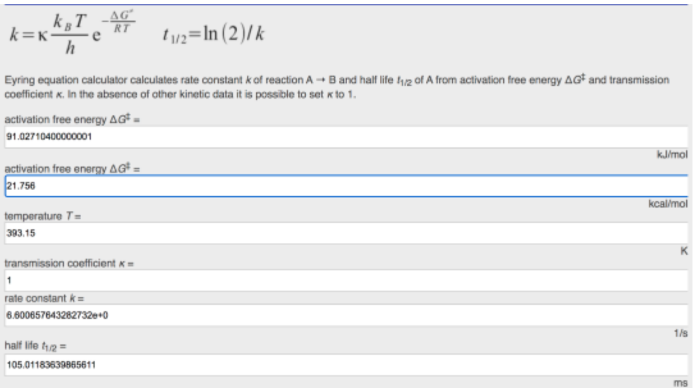

$$k=\kappa\frac{k_B T}{h}e^{-\frac{\Delta G^{\ddagger}}{RT}} \qquad t_{1/2}=\ln(2)/k$$

Eyring equation calculator calculates rate constant k of reaction A → B and half life $t_{1/2}$ of A from activation free energy $\Delta G^{\ddagger}$ and transmission coefficient κ. In the absence of other kinetic data it is possible to set κ to 1.

activation free energy $\Delta G^{\ddagger}$ =
91.02710400000001
kJ/mol

activation free energy $\Delta G^{\ddagger}$ =
21.756
kcal/mol

temperature T =
393.15
K

transmission coefficient κ =
1

rate constant k =
6.600657643282732e+0
1/s

half life $t_{1/2}$ =
105.01183639865611
ms

Simulated Spectrum

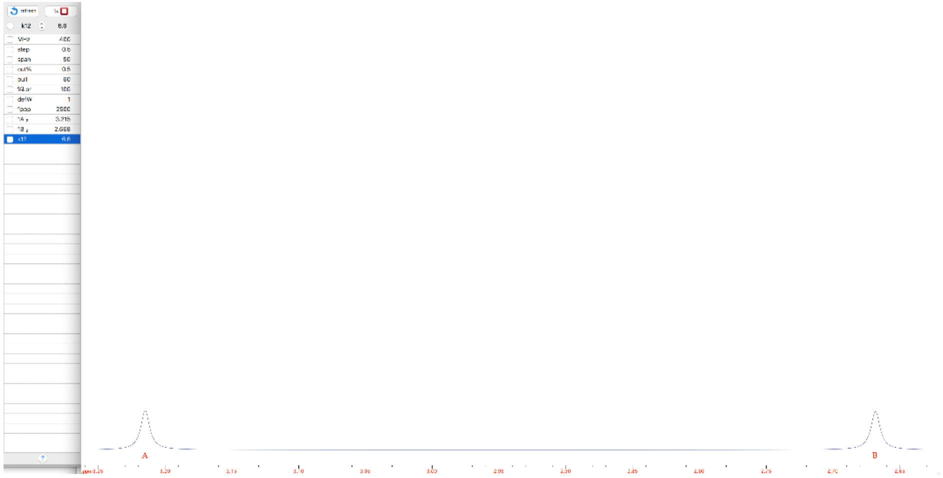

Experimental Spectrum

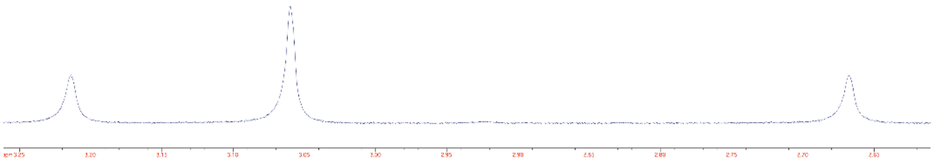

Enantiomerization – Thermodynamic Analysis of 3 at 55 °C, $CDCl_3$ solvent

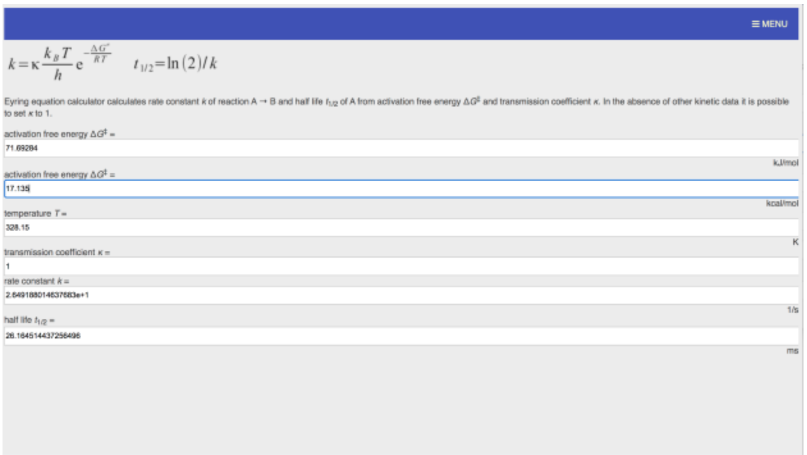

Coalescence Temperature: The coalescence temperature is defined as the temperature at which the appearance of the spectrum changes from that of two separate peaks to that of a single, flat-topped peak (see Figure 1C). At this temperature

$$k = \frac{\pi \Delta \nu_0}{\sqrt{2}} \tag{11}$$

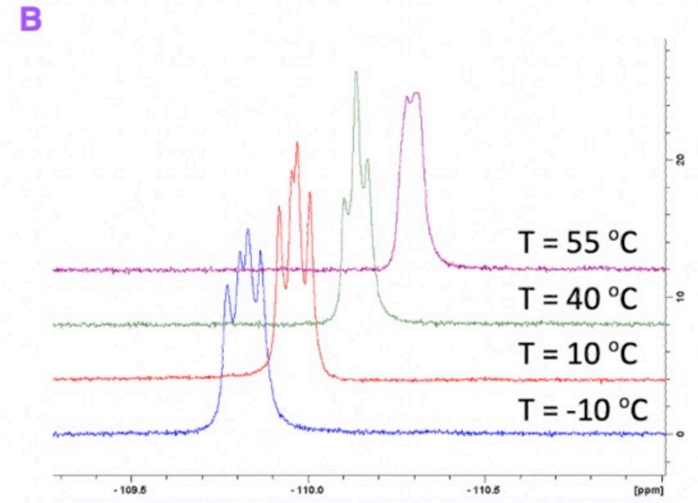

Computational Work

The Gaussian '09 package and Spartan '10 were used for all geometry optimizations,[9,10] which were determined using the M06-2X/6-311++G** level of theory. The calculated chemical shifts were fitted to the empirical equation at ωB97XD/6-311++G** $\delta_{calc} = -1.183\delta + 261.3$.[11]

Protocol for transition state searches. Transition states were found using a variety of DFT functionals; for consistency with the other parts of the paper M06-2X/6-311G** is our basic benchmark. Vibrational analysis confirmed the presence of saddle points. Following the lead of Miller and Rablen,[12] activation energies were approximated as electronic and zero point; vibrational corrections are noted to be unreliable for our types of systems due to the anharmonicity of amide rotational barriers. In practice, we found this to be true; although the trends remained the same, the absolute values of energies varied widely when vibrations were taken into account.

	Aldehyde 7	*Amide 3*	*Ester 4*	*Amide–(BBr_3)$_2$*
**F – C coupling**	19.3 Hz	7.2 Hz	2.9 Hz	3.2 Hz
**Probe F**	89.12 ppm	81.41 ppm	75.85 ppm	85.75 ppm
**Control F**	89.12 ppm	94.74 ppm	91.75 ppm	88.30 ppm

Table S5. *DFT calculated (ωB97XD/6-311++G**) NMR chemical shifts of the probe and control fluorine atoms and the coupling constants between probe fluorine and carbonyl atoms in Aldehyde 7, Amide 3, and Ester 4.*

	Amide 3	*Amide–(BBr_3)$_2$*
**Probe F**	-0.372 a.u.	-0.353 a.u.
**Control F**	-0.354 a.u.	-0.348 a.u.

Table S6. *DFT calculated (M06-2X/6-311++G**) NBO charges on the probe and control fluorine atoms of free amide* ***3*** *and double BBr_3 coordinated amide.*

[9] Gaussian 09, Revision A.1, M. J. Frisch, et al. Gaussian, Inc., Wallingford CT, 2009.
[10] Spartan ’10 Program, Wavefunction Inc., Irvine, CA.
[11] Holl, M. G.; Struble, M. D.; Singal, P.; Siegler, M. A.; Lectka, T. *Angew. Chem. Int. Ed.* **2016**, *55*, 8266-8269.
[12] Barrett, K. T.; Metrano, A. J.; Rablen, P. R.; Miller, S. J. *Nature* 2014, *509*, 71-75.

	Ground State	*Transition State*
**Amide 3**	-1232.495976	-1232.468156
**ΔE (Hartree)**	-0.02782	
**ΔE (kcal/mol)**	-17.45730038	
**Control Amide 8**	-1034.024099	-1033.991407
**ΔE (Hartree)**	-0.032692	
**ΔE (kcal/mol)**	-20.51452423	

Table S7. *DFT calculated (M06-2X/6-311++G**) barrier for amide isomerization in amide **3** and control amide **8**.*

	Energy (Hartree)	*ΔE relative to 3 (Hartree)*	*ΔE relative to 3 (kcal/mol)*
**3**	-1232.495976	0	0
**TS1**	-1232.468156	0.02782	17.46
**3a**	-1232.474763	0.021213	13.31
**TS2**	-1232.474712	0.021264	13.34
**3b**	-1232.493625	0.002351	1.48
**TS3**	-1232.488613	0.007363	4.62

Table S8. *DFT calculated (M06-2X/6-311++G**) energies for the structures in Figure **9**.*

	Probe Amide Protonation	*Control Amide Protonation*
**Energy**	-1232.850589	-1232.846227
**ΔE (Hartree)**	-0.004362	
**ΔE (kcal/mol)**	-2.737194258	

Table S9. *DFT calculated (M06-2X/6-311++G**) energies for the protonation of amide **3**.*

Coordinates of Calculated Structures

Equilibrium Geometry of 3 at M06-2X/6-311++G**

opt=noeigentest 6-311++g(d,p) scrf=(iefpcm,solvent=chloroform) geom=connectivity m062x

Center Number	Atomic Number	Atomic Type	Coordinates (Angstroms) X	Y	Z
1	6	0	-2.328237	-1.011857	0.246211
2	1	0	-1.931034	-3.106970	0.067009
3	6	0	-1.510896	-2.108214	0.114997
4	6	0	-0.377335	0.429529	0.357065
5	6	0	-0.110593	-1.964264	0.068029
6	6	0	-1.749031	0.253681	0.404283
7	6	0	0.478625	-0.675730	0.102408
8	6	0	0.719962	-3.138000	0.071826
9	1	0	-2.374880	1.113473	0.614709
10	6	0	2.064924	-3.039318	0.125898
11	1	0	0.233145	-4.107068	0.082169
12	1	0	2.686971	-3.923951	0.189680
13	6	0	2.713130	-1.760380	0.025361
14	6	0	4.117688	-1.691117	-0.054163
15	6	0	1.921765	-0.586251	-0.076935
16	6	0	4.751250	-0.496170	-0.300093
17	1	0	4.688812	-2.606046	0.059197
18	6	0	3.988527	0.656334	-0.527437
19	1	0	4.439704	1.600909	-0.801279
20	6	0	2.623089	0.582138	-0.433941
21	6	0	0.144418	1.744254	0.886256
22	8	0	0.881487	1.743472	1.853290
23	7	0	-0.351567	2.891528	0.331766
24	9	0	1.930826	1.684115	-0.787302
25	6	0	0.108496	4.144316	0.905536
26	1	0	-0.614425	4.926137	0.666342
27	1	0	1.088143	4.425147	0.499399
28	1	0	0.197673	4.040173	1.983244
29	6	0	-0.765953	2.973103	-1.060057
30	1	0	-1.669786	3.581100	-1.144299
31	1	0	-0.967633	1.984047	-1.461500
32	1	0	0.024484	3.434076	-1.661894
33	6	0	-3.850010	-1.170858	0.296597
34	8	0	-4.355152	-2.022455	1.026342
35	7	0	-4.695144	-0.317020	-0.514007
36	6	0	-4.090888	0.701977	-1.386299
37	1	0	-3.020090	0.499702	-1.496413
38	1	0	-4.230637	1.693968	-0.940373
39	1	0	-4.571963	0.673265	-2.370167
40	6	0	-6.157846	-0.469836	-0.466154
41	1	0	-6.408219	-1.488159	-0.148256
42	1	0	-6.577741	-0.282817	-1.460706
43	1	0	-6.576469	0.248573	0.248548
44	9	0	6.130700	-0.432480	-0.377487

Equilibrium Geometry for the NBO charge calculation of 3 at M06-2X/6-311++G**

opt=noeigentest freq 6-311++g(d,p) scrf=(iefpcm,solvent=chloroform) pop=(full,nbo) geom=connectivity m062x

Center Number	Atomic Number	Atomic Type	Coordinates (Angstroms) X	Y	Z
1	6	0	-2.439742	-0.304974	-0.259451
2	6	0	-1.824525	-1.529454	-0.356840
3	6	0	-0.426353	-1.647541	-0.261383
4	6	0	0.385794	-0.497618	-0.106840
5	6	0	-0.277466	0.742751	0.100706
6	6	0	-1.650861	0.823522	-0.009132
7	6	0	0.160488	-2.957691	-0.229595
8	6	0	1.832081	-0.688743	-0.122317
9	6	0	2.365382	-1.996276	0.015561
10	6	0	1.480446	-3.125790	-0.022671
11	6	0	3.752226	-2.215323	0.106820
12	1	0	4.145166	-3.215791	0.237205
13	6	0	4.603028	-1.151812	-0.005306
14	6	0	4.141500	0.134259	-0.265082
15	6	0	2.786527	0.323893	-0.340008
16	1	0	-0.496876	-3.815551	-0.323733
17	1	0	-2.412975	-2.430152	-0.500521
18	1	0	-2.140133	1.779918	0.137114
19	1	0	1.909053	-4.117861	0.064425
20	1	0	4.824122	0.955550	-0.435058
21	9	0	2.383395	1.547286	-0.721680
22	9	0	5.927599	-1.343751	0.082295
23	6	0	0.394851	1.937426	0.734970
24	6	0	-3.915745	-0.114163	-0.464888
25	8	0	0.969155	1.790143	1.800288
26	7	0	0.203944	3.155174	0.156923
27	6	0	0.758028	4.312826	0.833146
28	1	0	1.799266	4.485306	0.534348
29	1	0	0.165611	5.193614	0.574433
30	1	0	0.729679	4.154378	1.909027
31	6	0	-0.085302	3.340103	-1.253666
32	1	0	-0.898347	4.060739	-1.381176
33	1	0	0.803386	3.717489	-1.772391
34	1	0	-0.377880	2.401137	-1.716926
35	7	0	-4.769627	-0.934598	0.213919
36	6	0	-4.430868	-1.675664	1.416447
37	1	0	-3.395917	-1.505084	1.702000
38	1	0	-5.071583	-1.345446	2.241134
39	1	0	-4.586348	-2.749886	1.270066
40	6	0	-6.189732	-0.829082	-0.068402
41	1	0	-6.685901	-0.157746	0.642449
42	1	0	-6.331201	-0.435140	-1.072212
43	1	0	-6.645564	-1.819586	0.008528
44	8	0	-4.305565	0.732904	-1.251859

Equilibrium Geometry for the NMR calculations of 3 at ωB97XD/6-311++G**

nmr=(giao,spinspin) wb97xd/6-311++g(d,p) scrf=(iefpcm,solvent=dichloromethane) geom=connectivity

Center Number	Atomic Number	Atomic Type	Coordinates (Angstroms) X	Y	Z
1	6	0	-2.445288	-0.277156	-0.229994
2	6	0	-1.846810	-1.513474	-0.310869
3	6	0	-0.447895	-1.646004	-0.221630
4	6	0	0.375534	-0.500906	-0.081858
5	6	0	-0.271779	0.749395	0.117440
6	6	0	-1.644635	0.849331	0.006211
7	6	0	0.129397	-2.962576	-0.187696
8	6	0	1.819013	-0.701791	-0.110262
9	6	0	2.346494	-2.013223	0.021284
10	6	0	1.453622	-3.138724	0.001794
11	6	0	3.735300	-2.238074	0.087714
12	1	0	4.127965	-3.239518	0.210367
13	6	0	4.584808	-1.174525	-0.041479
14	6	0	4.128010	0.116760	-0.294204
15	6	0	2.771606	0.309876	-0.343323
16	1	0	-0.536266	-3.814580	-0.268877
17	1	0	-2.446543	-2.408641	-0.441918
18	1	0	-2.122306	1.813536	0.144524
19	1	0	1.877893	-4.132234	0.088612
20	1	0	4.811893	0.933927	-0.479493
21	9	0	2.358137	1.533879	-0.716186
22	9	0	5.911768	-1.370743	0.022963
23	6	0	0.427575	1.933281	0.741517
24	6	0	-3.918814	-0.071781	-0.443040
25	8	0	1.031411	1.770005	1.793676
26	7	0	0.245111	3.150841	0.176098
27	6	0	0.851252	4.295510	0.838769
28	1	0	1.909071	4.387028	0.567941
29	1	0	0.324185	5.197936	0.527529
30	1	0	0.773454	4.179118	1.916893
31	6	0	-0.048744	3.343862	-1.237269
32	1	0	-0.838139	4.089198	-1.355203
33	1	0	0.850505	3.695915	-1.753301
34	1	0	-0.368535	2.413064	-1.697471
35	7	0	-4.782321	-0.912232	0.177724
36	6	0	-4.474132	-1.714812	1.355102
37	1	0	-3.464533	-1.521092	1.705872
38	1	0	-5.171683	-1.450494	2.154256
39	1	0	-4.583952	-2.780650	1.137434
40	6	0	-6.199629	-0.793010	-0.134294
41	1	0	-6.689121	-0.078090	0.534746
42	1	0	-6.320113	-0.455135	-1.160233
43	1	0	-6.667351	-1.770820	-0.010939
44	8	0	-4.291963	0.816406	-1.201423

Equilibrium Geometry of 4 at M06-2X/6-311++G**

opt 6-311++g(d,p) scrf=(iefpcm,solvent=chloroform) geom=connectivity m062x

Center Number	Atomic Number	Atomic Type	Coordinates (Angstroms) X	Y	Z
1	6	0	-2.623081	-0.348667	-0.063872
2	6	0	-1.963641	-1.554096	0.014002
3	6	0	-0.558013	-1.600998	0.064679
4	6	0	0.204303	-0.409308	-0.015694
5	6	0	-0.505415	0.823513	0.023077
6	6	0	-1.884003	0.842472	-0.019568
7	6	0	0.093196	-2.864843	0.274599
8	6	0	1.651680	-0.537504	-0.086376
9	6	0	2.259337	-1.785340	0.212489
10	6	0	1.432884	-2.942821	0.415748
11	6	0	3.660274	-1.923304	0.235491
12	1	0	4.117980	-2.871577	0.487368
13	6	0	4.437438	-0.848854	-0.099705
14	6	0	3.894221	0.365321	-0.516162
15	6	0	2.529075	0.477525	-0.515324
16	1	0	-0.526417	-3.749950	0.363778
17	1	0	-2.523985	-2.480504	0.052113
18	1	0	-2.422209	1.781958	0.036981
19	1	0	1.917104	-3.887876	0.631692
20	1	0	4.518317	1.179578	-0.859001
21	9	0	2.018357	1.609536	-1.033455
22	9	0	5.773331	-0.965007	-0.082742
23	6	0	0.141321	2.121669	0.425043
24	6	0	-4.109403	-0.252273	-0.130825
25	8	0	0.786352	2.208476	1.434944
26	6	0	-0.105937	4.517401	0.305901
27	1	0	0.886970	4.961359	0.238404
28	1	0	-0.813661	5.111533	-0.267060
29	1	0	-0.425571	4.465996	1.346010
30	6	0	-6.132917	-1.434789	-0.185687
31	1	0	-6.544291	-0.900579	0.670235
32	1	0	-6.458626	-0.954307	-1.107865
33	1	0	-6.435235	-2.477737	-0.168570
34	8	0	-4.711401	0.790653	-0.195734
35	8	0	-4.701307	-1.446597	-0.116617
36	8	0	-0.116580	3.216310	-0.298244

Equilibrium Geometry for NMR calculations of 4 at ωB97XD/6-311++G**

nmr=(giao,spinspin) wb97xd/6-311++g(d,p) scrf=(iefpcm,solvent=chloroform) geom=connectivity

Center Number	Atomic Number	Atomic Type	Coordinates (Angstroms) X	Y	Z
1	6	0	-2.642040	-0.301821	-0.037498
2	6	0	-2.000704	-1.516539	0.058578
3	6	0	-0.596263	-1.582845	0.124911

4	6	0	0.178873	-0.400185	0.052138
5	6	0	-0.514197	0.840994	0.108684
6	6	0	-1.890213	0.881630	0.027685
7	6	0	0.044015	-2.853306	0.330409
8	6	0	1.621486	-0.544494	-0.054867
9	6	0	2.221191	-1.797503	0.234354
10	6	0	1.384776	-2.944665	0.458736
11	6	0	3.620951	-1.952371	0.214894
12	1	0	4.076305	-2.903686	0.458863
13	6	0	4.398121	-0.891190	-0.159262
14	6	0	3.856178	0.323616	-0.576730
15	6	0	2.493101	0.453959	-0.534560
16	1	0	-0.583376	-3.732925	0.419276
17	1	0	-2.574348	-2.435066	0.090483
18	1	0	-2.407428	1.832630	0.060044
19	1	0	1.861996	-3.894964	0.667460
20	1	0	4.480279	1.121933	-0.954682
21	9	0	1.978389	1.578010	-1.062913
22	9	0	5.733229	-1.022878	-0.185976
23	6	0	0.167982	2.110158	0.513769
24	6	0	-4.125081	-0.189084	-0.137528
25	8	0	0.985115	2.172479	1.397156
26	6	0	0.276290	4.429427	0.227838
27	1	0	1.356090	4.411133	0.080702
28	1	0	-0.187688	5.166085	-0.421440
29	1	0	0.053119	4.641563	1.273218
30	6	0	-6.156636	-1.350658	-0.282820
31	1	0	-6.585245	-0.846793	0.583130
32	1	0	-6.456150	-0.830602	-1.192344
33	1	0	-6.468549	-2.390520	-0.314582
34	8	0	-4.718767	0.859649	-0.179794
35	8	0	-4.727769	-1.378551	-0.179694
36	8	0	-0.288148	3.168206	-0.152982

Equilibrium Geometry of 7 at M06-2X/6-311++G**

opt 6-311++g(d,p) scrf=(iefpcm,solvent=chloroform) geom=connectivity m062x

Center Number	Atomic Number	Atomic Type	Coordinates (Angstroms) X	Y	Z
1	6	0	-3.162514	-0.561029	-0.139956
2	6	0	-2.331890	-1.661972	-0.128735
3	6	0	-0.937506	-1.516890	-0.007547
4	6	0	-0.370406	-0.220584	0.050631
5	6	0	-1.255117	0.888500	0.195313
6	6	0	-2.617796	0.715179	0.070750
7	6	0	-0.100951	-2.678122	0.123831
8	6	0	1.077429	-0.116475	-0.008313
9	6	0	1.871179	-1.274723	0.207931
10	6	0	1.231827	-2.557912	0.306265
11	6	0	3.276108	-1.193261	0.231796
12	1	0	3.878242	-2.072363	0.423153
13	6	0	3.874675	0.008109	-0.034913
14	6	0	3.148946	1.146166	-0.383965

15	6	0	1.782152	1.047145	-0.380757
16	1	0	-0.573196	-3.653892	0.115793
17	1	0	-2.751885	-2.661145	-0.201676
18	1	0	-3.277545	1.567022	0.192947
19	1	0	1.854219	-3.431091	0.462556
20	1	0	3.640449	2.062519	-0.681811
21	9	0	1.098399	2.111958	-0.836807
22	9	0	5.211590	0.099914	-0.017899
23	6	0	-0.808229	2.204927	0.741544
24	6	0	-4.626011	-0.746482	-0.279520
25	8	0	-1.458025	3.215737	0.658375
26	8	0	-5.422283	0.158841	-0.275623
27	1	0	0.128821	2.185226	1.323876
28	1	0	-4.957909	-1.795205	-0.390628

Equilibrium Geometry for NMR calculations of 7 at ωB97XD/6-311++G**

nmr=(giao,spinspin) wb97xd/6-311++g(d,p) scrf=(iefpcm,solvent=dichloromethane) geom=connectivity

Center Number	Atomic Number	Atomic Type	Coordinates (Angstroms) X	Y	Z
1	6	0	-3.163531	-0.560889	-0.137634
2	6	0	-2.332808	-1.661704	-0.129271
3	6	0	-0.938266	-1.516751	-0.009524
4	6	0	-0.370855	-0.220539	0.050187
5	6	0	-1.255771	0.888127	0.197259
6	6	0	-2.618509	0.715106	0.073606
7	6	0	-0.101979	-2.678427	0.119089
8	6	0	1.077176	-0.116729	-0.008173
9	6	0	1.870524	-1.275805	0.205662
10	6	0	1.230872	-2.558997	0.301209
11	6	0	3.275474	-1.195083	0.230236
12	1	0	3.877097	-2.074944	0.419697
13	6	0	3.874793	0.006596	-0.033211
14	6	0	3.149723	1.145845	-0.379533
15	6	0	1.782865	1.047465	-0.377131
16	1	0	-0.574519	-3.654040	0.109421
17	1	0	-2.752859	-2.660791	-0.203055
18	1	0	-3.278088	1.566991	0.196436
19	1	0	1.853047	-3.432691	0.455480
20	1	0	3.641700	2.062774	-0.674792
21	9	0	1.100203	2.113982	-0.830706
22	9	0	5.211759	0.097636	-0.015498
23	6	0	-0.808609	2.206004	0.739464
24	6	0	-4.627143	-0.746229	-0.275830
25	8	0	-1.451623	3.219687	0.640975
26	8	0	-5.423515	0.159008	-0.269608
27	1	0	0.122408	2.185454	1.331481
28	1	0	-4.959065	-1.794810	-0.388204

Equilibrium Geometry of Amide–$(BBr_3)_2$ at M06-2X/6-311++G**

opt=noeigentest 6-311++g(d,p) scrf=(iefpcm,solvent=chloroform) geom=connectivity m062x

Center Number	Atomic Number	Atomic Type	Coordinates (Angstroms) X	Y	Z
1	6	0	-1.335252	-0.008535	-1.015747
2	6	0	-0.985274	1.066943	-1.797309
3	6	0	0.161249	1.823857	-1.499841
4	6	0	0.929550	1.547465	-0.341556
5	6	0	0.568607	0.389517	0.403208
6	6	0	-0.536253	-0.369658	0.071824
7	6	0	0.596359	2.811092	-2.446914
8	6	0	2.067388	2.411577	-0.063640
9	6	0	2.531314	3.293544	-1.076367
10	6	0	1.768020	3.457382	-2.280935
11	6	0	3.689130	4.070862	-0.891334
12	1	0	4.045929	4.724758	-1.676770
13	6	0	4.342183	4.013675	0.308944
14	6	0	3.873097	3.250948	1.376151
15	6	0	2.751165	2.495490	1.164636
16	1	0	-0.014164	2.981403	-3.325815
17	1	0	-1.573231	1.326700	-2.671187
18	1	0	-0.762243	-1.269958	0.633116
19	1	0	2.132605	4.152333	-3.027963
20	1	0	4.353199	3.273761	2.344992
21	9	0	2.252604	1.851246	2.237266
22	9	0	5.446321	4.746696	0.499888
23	6	0	1.475112	-0.275130	1.365230
24	6	0	-2.543058	-0.802953	-1.338056
25	8	0	2.678305	-0.599484	0.971568
26	7	0	1.111169	-0.596098	2.566658
27	6	0	2.002023	-1.368703	3.442989
28	1	0	2.595963	-0.672709	4.038374
29	1	0	1.378755	-1.974929	4.097686
30	1	0	2.651934	-2.004413	2.850402
31	6	0	-0.107737	-0.087536	3.207468
32	1	0	-0.798616	-0.915305	3.371583
33	1	0	0.181537	0.339563	4.168180
34	1	0	-0.580450	0.673589	2.593498
35	7	0	-2.459681	-2.016603	-1.788306
36	6	0	-1.190959	-2.666141	-2.148578
37	1	0	-0.375941	-1.947200	-2.169547
38	1	0	-0.975596	-3.446591	-1.418251
39	1	0	-1.316732	-3.113417	-3.134527
40	6	0	-3.668837	-2.823803	-2.009527
41	1	0	-3.416146	-3.860126	-1.793926
42	1	0	-4.465204	-2.483336	-1.354560
43	1	0	-3.975186	-2.728811	-3.052141
44	8	0	-3.728196	-0.279349	-1.203952
45	5	0	2.983595	-1.557313	-0.099210
46	35	0	4.920917	-2.040196	0.142123
47	35	0	1.782462	-3.205259	0.170489
48	35	0	2.663058	-0.818205	-1.965425
49	5	0	-4.236121	0.327607	0.038201
50	35	0	-6.235542	0.406262	-0.171539

51	35	0	-3.727724	-0.925878	1.584011
52	35	0	-3.477523	2.173332	0.383892

Equilibrium Geometry for NMR calculations of Amide–$(BBr_3)_2$ at ωB97XD/6-311++G**

nmr=(giao,spinspin) wb97xd/6-311++g(d,p) scrf=(iefpcm,solvent=dichloromethane) geom=connectivity

----

Center Number	Atomic Number	Atomic Type	Coordinates (Angstroms) X	Y	Z
1	6	0	-1.356242	-0.039798	-1.008982
2	6	0	-1.009497	1.038801	-1.787849
3	6	0	0.135281	1.797994	-1.488840
4	6	0	0.905530	1.520097	-0.332051
5	6	0	0.551952	0.355564	0.406295
6	6	0	-0.551645	-0.405650	0.073528
7	6	0	0.565275	2.791010	-2.432460
8	6	0	2.039327	2.388881	-0.051035
9	6	0	2.498176	3.277659	-1.060382
10	6	0	1.733849	3.442574	-2.264282
11	6	0	3.652134	4.060308	-0.872664
12	1	0	4.004649	4.719547	-1.655631
13	6	0	4.305668	4.001558	0.327324
14	6	0	3.841216	3.232090	1.391789
15	6	0	2.723147	2.471544	1.177476
16	1	0	-0.046381	2.961736	-3.310501
17	1	0	-1.599431	1.300448	-2.659797
18	1	0	-0.774192	-1.308742	0.631853
19	1	0	2.094542	4.142632	-3.008427
20	1	0	4.320914	3.254001	2.360929
21	9	0	2.228639	1.820302	2.247404
22	9	0	5.406480	4.739625	0.520788
23	6	0	1.463091	-0.307388	1.365908
24	6	0	-2.567499	-0.830863	-1.329052
25	8	0	2.680819	-0.591718	0.981671
26	7	0	1.095256	-0.655368	2.558128
27	6	0	1.994203	-1.420255	3.433224
28	1	0	2.564906	-0.719497	4.045332
29	1	0	1.377835	-2.049808	4.072055
30	1	0	2.664915	-2.032310	2.839144
31	6	0	-0.141576	-0.185697	3.194078
32	1	0	-0.813955	-1.032249	3.337270
33	1	0	0.128395	0.231742	4.164485
34	1	0	-0.624562	0.574706	2.587393
35	7	0	-2.489389	-2.043918	-1.779999
36	6	0	-1.223791	-2.697187	-2.143288
37	1	0	-0.407268	-1.980152	-2.164872
38	1	0	-1.010974	-3.479507	-1.414305
39	1	0	-1.353328	-3.142021	-3.129703
40	6	0	-3.701054	-2.846689	-2.004017
41	1	0	-3.451446	-3.884398	-1.792108
42	1	0	-4.496411	-2.506421	-1.347930
43	1	0	-4.006262	-2.746887	-3.046407
44	8	0	-3.751600	-0.300859	-1.197304
45	5	0	3.032985	-1.516782	-0.102010

46	35	0	4.983397	-1.941513	0.163932
47	35	0	1.885759	-3.209597	0.107601
48	35	0	2.725998	-0.749087	-1.959615
49	5	0	-4.262261	0.314627	0.037966
50	35	0	-6.262824	0.398028	-0.182409
51	35	0	-3.768395	-0.923926	1.599136
52	35	0	-3.505698	2.164294	0.374428

Equilibrium Geometry for NBO charge calculations of Amide–$(BBr_3)_2$ at M06-2X/6-311++G**

opt freq 6-311++g(d,p) scrf=(iefpcm,solvent=chloroform) pop=nbo geom=connectivity m062x

Center Number	Atomic Number	Atomic Type	Coordinates (Angstroms) X	Y	Z
1	6	0	-1.335252	-0.008535	-1.015747
2	6	0	-0.985274	1.066943	-1.797309
3	6	0	0.161249	1.823857	-1.499841
4	6	0	0.929550	1.547465	-0.341556
5	6	0	0.568607	0.389517	0.403208
6	6	0	-0.536253	-0.369658	0.071824
7	6	0	0.596359	2.811092	-2.446914
8	6	0	2.067388	2.411577	-0.063640
9	6	0	2.531314	3.293544	-1.076367
10	6	0	1.768020	3.457382	-2.280935
11	6	0	3.689130	4.070862	-0.891334
12	1	0	4.045929	4.724758	-1.676770
13	6	0	4.342183	4.013675	0.308944
14	6	0	3.873097	3.250948	1.376151
15	6	0	2.751165	2.495490	1.164636
16	1	0	-0.014164	2.981403	-3.325815
17	1	0	-1.573231	1.326700	-2.671187
18	1	0	-0.762243	-1.269958	0.633116
19	1	0	2.132605	4.152333	-3.027963
20	1	0	4.353199	3.273761	2.344992
21	9	0	2.252604	1.851246	2.237266
22	9	0	5.446321	4.746696	0.499888
23	6	0	1.475112	-0.275130	1.365230
24	6	0	-2.543058	-0.802953	-1.338056
25	8	0	2.678305	-0.599484	0.971568
26	7	0	1.111169	-0.596098	2.566658
27	6	0	2.002023	-1.368703	3.442989
28	1	0	2.595963	-0.672709	4.038374
29	1	0	1.378755	-1.974929	4.097686
30	1	0	2.651934	-2.004413	2.850402
31	6	0	-0.107737	-0.087536	3.207468
32	1	0	-0.798616	-0.915305	3.371583
33	1	0	0.181537	0.339563	4.168180
34	1	0	-0.580450	0.673589	2.593498
35	7	0	-2.459681	-2.016603	-1.788306
36	6	0	-1.190959	-2.666141	-2.148578
37	1	0	-0.375941	-1.947200	-2.169547
38	1	0	-0.975596	-3.446591	-1.418251

39	1	0	-1.316732	-3.113417	-3.134527
40	6	0	-3.668837	-2.823803	-2.009527
41	1	0	-3.416146	-3.860126	-1.793926
42	1	0	-4.465204	-2.483336	-1.354560
43	1	0	-3.975186	-2.728811	-3.052141
44	8	0	-3.728196	-0.279349	-1.203952
45	5	0	2.983595	-1.557313	-0.099210
46	35	0	4.920917	-2.040196	0.142123
47	35	0	1.782462	-3.205259	0.170489
48	35	0	2.663058	-0.818205	-1.965425
49	5	0	-4.236121	0.327607	0.038201
50	35	0	-6.235542	0.406262	-0.171539
51	35	0	-3.727724	-0.925878	1.584011
52	35	0	-3.477523	2.173332	0.383892

Equilibrium Geometry for the Protonation of Probe Amide in 3 at M06-2X/6-311++G**

opt 6-311++g(d,p) geom=connectivity m062x

Center Number	Atomic Number	Atomic Type	Coordinates (Angstroms) X	Y	Z
1	6	0	-2.332613	-0.978380	0.150361
2	1	0	-1.932985	-3.068699	0.124202
3	6	0	-1.500840	-2.073934	0.112448
4	6	0	-0.382743	0.457910	0.261174
5	6	0	-0.100840	-1.936382	0.083038
6	6	0	-1.758431	0.292372	0.273937
7	6	0	0.497168	-0.651018	0.077578
8	6	0	0.716750	-3.116730	0.126657
9	1	0	-2.399315	1.149888	0.453089
10	6	0	2.062058	-3.029482	0.176864
11	1	0	0.220015	-4.079171	0.157763
12	1	0	2.675291	-3.918856	0.259843
13	6	0	2.719604	-1.760381	0.044074
14	6	0	4.123566	-1.720533	-0.040973
15	6	0	1.943102	-0.575759	-0.081945
16	6	0	4.740672	-0.529975	-0.312207
17	1	0	4.707886	-2.624483	0.073651
18	6	0	4.025431	0.642486	-0.552329
19	1	0	4.520448	1.559300	-0.842935
20	6	0	2.662604	0.580251	-0.447099
21	6	0	0.084450	1.772870	0.743171
22	8	0	0.890381	1.815841	1.780314
23	7	0	-0.310663	2.912726	0.270297
24	9	0	1.981520	1.696410	-0.775812
25	6	0	0.071856	4.170827	0.927943
26	1	0	-0.707429	4.901593	0.721931
27	1	0	1.021063	4.516649	0.515115
28	1	0	0.166618	4.016525	1.998390
29	6	0	-0.980922	3.058321	-1.027108
30	1	0	-2.002154	3.407477	-0.872952
31	1	0	-0.977896	2.116117	-1.566111
32	1	0	-0.426486	3.804280	-1.597117

33	6	0	-3.819942	-1.198061	0.261258
34	8	0	-4.232638	-2.088203	0.991408
35	7	0	-4.635402	-0.358531	-0.418304
36	6	0	-4.240705	0.445824	-1.568539
37	1	0	-3.208043	0.251111	-1.847684
38	1	0	-4.369953	1.511099	-1.358768
39	1	0	-4.873533	0.180990	-2.419053
40	6	0	-6.073236	-0.483537	-0.212558
41	1	0	-6.267197	-0.823857	0.801256
42	1	0	-6.507103	-1.198861	-0.917496
43	1	0	-6.533103	0.492987	-0.368409
44	9	0	6.073039	-0.477589	-0.400890
45	1	0	1.021435	0.935926	2.163401

Equilibrium Geometry for the Protonation of Control Amide in 3 at M06-2X/6-311++G**

opt 6-311++g(d,p) geom=connectivity m062x

Center Number	Atomic Number	Atomic Type	Coordinates (Angstroms) X	Y	Z
1	6	0	-2.312413	-0.925421	0.160813
2	1	0	-1.953342	-3.042368	-0.000106
3	6	0	-1.517595	-2.050676	0.072955
4	6	0	-0.346360	0.477185	0.311904
5	6	0	-0.118040	-1.930262	0.046077
6	6	0	-1.716796	0.337825	0.323642
7	6	0	0.491958	-0.649243	0.073484
8	6	0	0.685009	-3.121940	0.057582
9	1	0	-2.335095	1.206060	0.526947
10	6	0	2.030073	-3.046391	0.122535
11	1	0	0.180565	-4.080994	0.058947
12	1	0	2.634601	-3.943231	0.190370
13	6	0	2.700578	-1.779154	0.027725
14	6	0	4.105657	-1.748227	-0.031858
15	6	0	1.934948	-0.588653	-0.086701
16	6	0	4.730957	-0.554228	-0.267759
17	1	0	4.684305	-2.656481	0.076320
18	6	0	4.025981	0.624266	-0.506118
19	1	0	4.532233	1.541367	-0.775329
20	6	0	2.659839	0.570460	-0.430173
21	6	0	0.183835	1.782346	0.866663
22	8	0	0.891236	1.740740	1.861316
23	7	0	-0.266235	2.931373	0.313361
24	9	0	1.993867	1.680353	-0.788486
25	6	0	0.159740	4.182546	0.926409
26	1	0	-0.545562	4.964870	0.645537
27	1	0	1.161098	4.461703	0.582542
28	1	0	0.175466	4.074317	2.007956
29	6	0	-0.709834	3.037217	-1.070532
30	1	0	-1.684205	3.529618	-1.120341
31	1	0	-0.775809	2.054878	-1.531535
32	1	0	0.012891	3.630267	-1.638587
33	6	0	-3.775341	-1.067027	0.199014

34	8	0	-4.317820	-2.011583	0.933367
35	7	0	-4.595990	-0.305088	-0.457710
36	6	0	-4.171593	0.639543	-1.500818
37	1	0	-3.162468	0.414557	-1.832394
38	1	0	-4.230851	1.658221	-1.116854
39	1	0	-4.861055	0.527448	-2.336625
40	6	0	-6.050722	-0.418912	-0.268229
41	1	0	-6.268782	-0.774170	0.734041
42	1	0	-6.455800	-1.112267	-1.006143
43	1	0	-6.478854	0.570057	-0.418284
44	9	0	6.066650	-0.506960	-0.329968
45	1	0	-3.665015	-2.429155	1.512923

Energy Calculations for Structures in Figure 9

Equilibrium Geometry of TS1 at M06-2X/6-311++G** (Amide Isomerization)

opt=(calcfc,ts,noeigentest) 6-311++g(d,p) scrf=(iefpcm,solvent=chloroform) geom=connectivity m062x

Center Number	Atomic Number	Atomic Type	Coordinates (Angstroms) X	Y	Z
1	6	0	2.246242	-1.082434	-0.190991
2	1	0	1.769064	-3.158156	-0.395771
3	6	0	1.387323	-2.147568	-0.305136
4	6	0	0.358808	0.445603	-0.233411
5	6	0	-0.010850	-1.953378	-0.302833
6	6	0	1.723952	0.218781	-0.190445
7	6	0	-0.547446	-0.652423	-0.154988
8	6	0	-0.886123	-3.073360	-0.515812
9	1	0	2.385449	1.075344	-0.196800
10	6	0	-2.225359	-2.909056	-0.565502
11	1	0	-0.438215	-4.047699	-0.679604
12	1	0	-2.882387	-3.743667	-0.784670
13	6	0	-2.820027	-1.643957	-0.232959
14	6	0	-4.220079	-1.537039	-0.104652
15	6	0	-1.981762	-0.539894	0.061271
16	6	0	-4.791941	-0.388648	0.387334
17	1	0	-4.833614	-2.389474	-0.371229
18	6	0	-3.975151	0.666772	0.816834
19	1	0	-4.381332	1.552444	1.289070
20	6	0	-2.616305	0.562777	0.667659
21	6	0	-0.081667	1.813840	-0.656709
22	8	0	-1.103938	2.003476	-1.262780
23	7	0	0.854289	2.880914	-0.390211
24	9	0	-1.864724	1.538262	1.210610
25	6	0	1.473596	3.315953	-1.645629
26	1	0	2.237096	4.060989	-1.416265
27	1	0	0.739480	3.752708	-2.335546
28	1	0	1.951159	2.467289	-2.138580
29	6	0	0.167459	3.997773	0.264247
30	1	0	0.911687	4.759340	0.502114
31	1	0	-0.284677	3.650818	1.193165
32	1	0	-0.612515	4.437593	-0.369860
33	6	0	3.760998	-1.300428	-0.145163
34	8	0	4.285403	-2.129220	-0.887746
35	7	0	4.577879	-0.529114	0.771937
36	6	0	6.032974	-0.738141	0.814586
37	1	0	6.248044	-1.812643	0.800923
38	1	0	6.438458	-0.296463	1.732398
39	1	0	6.496469	-0.261243	-0.056265
40	6	0	3.949754	0.462932	1.658963
41	1	0	4.332161	0.334587	2.677737
42	1	0	2.863437	0.320774	1.654952
43	1	0	4.187376	1.472111	1.304202
44	9	0	-6.165321	-0.287142	0.518521

Equilibrium Geometry for Frequency Calculations of TS1 at M06-2X/6-311++G** (Amide Isomerization)

freq 6-311++g(d,p) scrf=(iefpcm,solvent=chloroform) geom=connectivity m062x

Number of imaginary frequencies = 1

Center Number	Atomic Number	Atomic Type	Coordinates (Angstroms) X	Y	Z
1	6	0	2.265760	-1.030695	-0.124766
2	1	0	1.836820	-3.102191	-0.378880
3	6	0	1.420846	-2.106472	-0.269192
4	6	0	0.355035	0.469670	-0.206715
5	6	0	0.022350	-1.934642	-0.276538
6	6	0	1.720746	0.263871	-0.140561
7	6	0	-0.536004	-0.642496	-0.134623
8	6	0	-0.833857	-3.068763	-0.492878
9	1	0	2.373558	1.128138	-0.160422
10	6	0	-2.175947	-2.927271	-0.550454
11	1	0	-0.370743	-4.036596	-0.649061
12	1	0	-2.817163	-3.772899	-0.770092
13	6	0	-2.791809	-1.669364	-0.230656
14	6	0	-4.194796	-1.588748	-0.119898
15	6	0	-1.971988	-0.548901	0.060158
16	6	0	-4.753576	-0.433395	0.350567
17	1	0	-4.819085	-2.437152	-0.370174
18	6	0	-3.986525	0.650458	0.778977
19	1	0	-4.440798	1.520368	1.233588
20	6	0	-2.627292	0.554869	0.642670
21	6	0	-0.090822	1.827300	-0.655504
22	8	0	-1.086619	1.986029	-1.315240
23	7	0	0.802052	2.914281	-0.343028
24	9	0	-1.896026	1.548174	1.177480
25	6	0	1.413560	3.421278	-1.579309
26	1	0	2.137537	4.193079	-1.314286
27	1	0	0.667069	3.847823	-2.261748
28	1	0	1.938488	2.613120	-2.092819
29	6	0	0.065163	3.984005	0.340740
30	1	0	0.775338	4.766893	0.610328
31	1	0	-0.386837	3.593611	1.252718
32	1	0	-0.720182	4.414388	-0.293420
33	6	0	3.750334	-1.271068	-0.141030
34	8	0	4.219679	-2.112878	-0.898441
35	7	0	4.525189	-0.501389	0.662980
36	6	0	5.971221	-0.630473	0.552473
37	1	0	6.348611	-1.411592	1.220126
38	1	0	6.426991	0.321309	0.828806
39	1	0	6.238664	-0.881764	-0.470719
40	6	0	4.056400	0.224710	1.837385
41	1	0	4.640133	-0.095552	2.704292
42	1	0	3.009532	0.013515	2.037483
43	1	0	4.188660	1.302316	1.708100
44	9	0	-6.087814	-0.338785	0.465952

Equilibrium Geometry of 3a at M06-2X/6-311++G**

opt 6-311++g(d,p) scrf=(iefpcm,solvent=chloroform) geom=connectivity m062x

Center Number	Atomic Number	Atomic Type	Coordinates (Angstroms) X	Y	Z
1	6	0	4.757613	-0.435835	0.421045
2	1	0	4.869675	-2.091645	-0.921698
3	6	0	4.221829	-1.380038	-0.421782
4	6	0	2.599980	0.343229	1.032287
5	6	0	2.823521	-1.445253	-0.563709
6	6	0	3.963486	0.408240	1.201505
7	6	0	1.991535	-0.479444	0.059381
8	6	0	2.206273	-2.567889	-1.223161
9	1	0	4.399029	1.054397	1.951718
10	6	0	0.870644	-2.763946	-1.137955
11	1	0	2.849266	-3.303908	-1.697203
12	1	0	0.413322	-3.668430	-1.529902
13	6	0	0.002699	-1.752864	-0.588230
14	6	0	-1.387829	-1.947805	-0.536382
15	6	0	0.558532	-0.519184	-0.160092
16	6	0	-2.224557	-0.940495	-0.096673
17	1	0	-1.820611	-2.895901	-0.843791
18	6	0	-1.688695	0.346874	0.073791
19	1	0	-2.362283	1.187289	0.220488
20	6	0	-0.328114	0.591109	-0.036837
21	9	0	1.833749	0.981258	1.912587
22	6	0	0.140414	2.006992	-0.277178
23	8	0	1.255540	2.417032	-0.009964
24	7	0	-0.800327	2.861982	-0.839534
25	6	0	-1.664838	2.484181	-1.953980
26	1	0	-1.300158	2.957720	-2.875238
27	1	0	-2.692658	2.819966	-1.779109
28	1	0	-1.671679	1.407075	-2.108983
29	6	0	-0.390617	4.257859	-0.895015
30	1	0	0.073274	4.542365	0.047891
31	1	0	-1.278557	4.870834	-1.069614
32	1	0	0.333507	4.434536	-1.702140
33	6	0	-3.700702	-1.215770	-0.053620
34	8	0	-4.202933	-1.979662	-0.864695
35	7	0	-4.450565	-0.537613	0.873870
36	6	0	-3.944957	-0.044945	2.144668
37	1	0	-4.404034	-0.607542	2.966132
38	1	0	-4.191801	1.014023	2.275177
39	1	0	-2.865376	-0.168205	2.210201
40	6	0	-5.890539	-0.713377	0.819715
41	1	0	-6.214810	-1.538368	1.465585
42	1	0	-6.184578	-0.933354	-0.203791
43	1	0	-6.373981	0.208650	1.157912
44	9	0	6.084448	-0.364467	0.570369

Equilibrium Geometry of TS2 at M06-2X/6-311++G**

opt=(calcfc,ts,noeigentest) 6-311++g(d,p) scrf=(iefpcm,solvent=chloroform) geom=connectivity m062x

Center Number	Atomic Number	Atomic Type	Coordinates (Angstroms) X	Y	Z
1	6	0	2.263033	-0.868221	-0.098740
2	1	0	1.879698	-2.806455	-0.909022
3	6	0	1.442050	-1.878309	-0.557495
4	6	0	0.338509	0.609521	0.062678
5	6	0	0.047853	-1.707806	-0.588300
6	6	0	1.705013	0.399360	0.126346
7	6	0	-0.525007	-0.508208	-0.098633
8	6	0	-0.804433	-2.692915	-1.205841
9	1	0	2.363115	1.251246	0.266333
10	6	0	-2.136322	-2.491851	-1.317235
11	1	0	-0.338441	-3.571906	-1.636882
12	1	0	-2.763202	-3.195338	-1.852801
13	6	0	-2.772494	-1.409718	-0.609277
14	6	0	-4.174809	-1.351189	-0.505753
15	6	0	-1.961555	-0.494722	0.105329
16	6	0	-4.728056	-0.471160	0.387197
17	1	0	-4.805585	-2.020400	-1.076974
18	6	0	-3.962687	0.300482	1.260038
19	1	0	-4.421057	0.875323	2.053351
20	6	0	-2.596852	0.242294	1.123870
21	6	0	-0.153455	2.028573	-0.133226
22	8	0	-1.128792	2.508084	0.419908
23	7	0	0.597666	2.773141	-1.003382
24	9	0	-1.855613	0.779706	2.095696
25	6	0	1.366060	2.247126	-2.130672
26	1	0	2.427449	2.489060	-2.029497
27	1	0	0.990650	2.705129	-3.049726
28	1	0	1.255018	1.169856	-2.220709
29	6	0	0.253216	4.183624	-1.124322
30	1	0	1.123271	4.717921	-1.507791
31	1	0	-0.020038	4.582237	-0.150454
32	1	0	-0.586411	4.329686	-1.812083
33	6	0	3.749542	-1.093256	-0.092230
34	8	0	4.270176	-1.738290	-0.995881
35	7	0	4.472280	-0.521482	0.902785
36	6	0	3.937067	-0.084437	2.186871
37	1	0	2.878119	-0.311768	2.268566
38	1	0	4.087052	0.989842	2.323761
39	1	0	4.462973	-0.613186	2.986235
40	6	0	5.923421	-0.622704	0.842406
41	1	0	6.274347	-1.535209	1.334916
42	1	0	6.353742	0.241326	1.350466
43	1	0	6.246118	-0.636453	-0.195520
44	9	0	-6.065284	-0.401904	0.501164

Equilibrium Geometry for Frequency Calculations of TS2 at M06-2X/6-311++G**

freq 6-311++g(d,p) scrf=(iefpcm,solvent=chloroform) geom=connectivity m062x

Number of imaginary frequencies = 1

Center Number	Atomic Number	Atomic Type	Coordinates (Angstroms) X	Y	Z
1	6	0	2.263033	-0.868221	-0.098740
2	1	0	1.879698	-2.806455	-0.909022
3	6	0	1.442050	-1.878309	-0.557495
4	6	0	0.338509	0.609521	0.062678
5	6	0	0.047853	-1.707806	-0.588300
6	6	0	1.705013	0.399360	0.126346
7	6	0	-0.525007	-0.508208	-0.098633
8	6	0	-0.804433	-2.692915	-1.205841
9	1	0	2.363115	1.251246	0.266333
10	6	0	-2.136322	-2.491851	-1.317235
11	1	0	-0.338441	-3.571906	-1.636882
12	1	0	-2.763202	-3.195338	-1.852801
13	6	0	-2.772494	-1.409718	-0.609277
14	6	0	-4.174809	-1.351189	-0.505753
15	6	0	-1.961555	-0.494722	0.105329
16	6	0	-4.728056	-0.471160	0.387197
17	1	0	-4.805585	-2.020400	-1.076974
18	6	0	-3.962687	0.300482	1.260038
19	1	0	-4.421057	0.875323	2.053351
20	6	0	-2.596852	0.242294	1.123870
21	6	0	-0.153455	2.028573	-0.133226
22	8	0	-1.128792	2.508084	0.419908
23	7	0	0.597666	2.773141	-1.003382
24	9	0	-1.855613	0.779706	2.095696
25	6	0	1.366060	2.247126	-2.130672
26	1	0	2.427449	2.489060	-2.029497
27	1	0	0.990650	2.705129	-3.049726
28	1	0	1.255018	1.169856	-2.220709
29	6	0	0.253216	4.183624	-1.124322
30	1	0	1.123271	4.717921	-1.507791
31	1	0	-0.020038	4.582237	-0.150454
32	1	0	-0.586411	4.329686	-1.812083
33	6	0	3.749542	-1.093256	-0.092230
34	8	0	4.270176	-1.738290	-0.995881
35	7	0	4.472280	-0.521482	0.902785
36	6	0	3.937067	-0.084437	2.186871
37	1	0	2.878119	-0.311768	2.268566
38	1	0	4.087052	0.989842	2.323761
39	1	0	4.462973	-0.613186	2.986235
40	6	0	5.923421	-0.622704	0.842406
41	1	0	6.274347	-1.535209	1.334916
42	1	0	6.353742	0.241326	1.350466
43	1	0	6.246118	-0.636453	-0.195520
44	9	0	-6.065284	-0.401904	0.501164

Equilibrium Geometry of 3b at M06-2X/6-311++G**

opt=noeigentest 6-311++g(d,p) scrf=(iefpcm,solvent=chloroform) geom=connectivity m062x

Center Number	Atomic Number	Atomic Type	Coordinates (Angstroms) X	Y	Z
1	6	0	-2.573339	-0.151014	-0.378413
2	6	0	-1.994995	-1.390900	-0.206655
3	6	0	-0.596383	-1.535618	-0.125204
4	6	0	0.260563	-0.401132	-0.243237
5	6	0	-0.364438	0.880239	-0.280547
6	6	0	-1.742029	0.978474	-0.403046
7	6	0	-0.048534	-2.830874	0.159963
8	6	0	1.699228	-0.650282	-0.264908
9	6	0	2.199549	-1.933611	0.115539
10	6	0	1.279529	-3.008952	0.345085
11	6	0	3.584830	-2.184669	0.192194
12	1	0	3.950993	-3.154903	0.503125
13	6	0	4.463507	-1.198314	-0.170345
14	6	0	4.033253	0.030780	-0.663223
15	6	0	2.677857	0.261175	-0.716827
16	1	0	-0.735561	-3.663046	0.267853
17	1	0	-2.611800	-2.280546	-0.132302
18	1	0	-2.183219	1.963111	-0.500840
19	1	0	1.678147	-3.979527	0.617154
20	1	0	4.731064	0.771246	-1.030278
21	9	0	2.295904	1.410439	-1.300893
22	9	0	5.791377	-1.425906	-0.098260
23	6	0	0.315845	2.224460	-0.123522
24	6	0	-4.049060	0.026271	-0.619277
25	8	0	0.099893	3.121100	-0.924422
26	7	0	1.136708	2.387245	0.960944
27	6	0	1.845113	3.651863	1.105337
28	1	0	1.333723	4.312743	1.815089
29	1	0	2.855482	3.457457	1.475220
30	1	0	1.894623	4.147734	0.139504
31	6	0	1.187059	1.512083	2.122289
32	1	0	0.510964	0.669279	2.006735
33	1	0	2.201770	1.130660	2.277177
34	1	0	0.889713	2.069713	3.017660
35	7	0	-4.921018	-0.579559	0.245459
36	6	0	-4.590643	-1.001705	1.599872
37	1	0	-3.531508	-0.866676	1.801115
38	1	0	-5.158159	-0.404627	2.322569
39	1	0	-4.848690	-2.055028	1.750054
40	6	0	-6.346671	-0.509309	-0.050539
41	1	0	-6.822863	0.316517	0.490771
42	1	0	-6.486149	-0.352773	-1.116988
43	1	0	-6.820732	-1.445947	0.252960
44	8	0	-4.422971	0.658428	-1.597607

Equilibrium Geometry of TS3 at M06-2X/6-311++G**

opt=(calcfc,ts,noeigentest) 6-311++g(d,p) scrf=(iefpcm,solvent=chloroform) geom=connectivity m062x

Center Number	Atomic Number	Atomic Type	Coordinates (Angstroms) X	Y	Z
1	6	0	-4.418822	-1.356622	-0.088971
2	6	0	-3.551201	-2.394874	0.079501
3	6	0	-2.168245	-2.143076	0.123824
4	6	0	-1.707710	-0.832442	-0.050369
5	6	0	-2.621177	0.204711	-0.206218
6	6	0	-3.945370	-0.044912	-0.217366
7	6	0	-1.244997	-3.177197	0.346765
8	6	0	-0.337365	-0.559954	-0.066857
9	6	0	0.569713	-1.602475	0.124708
10	6	0	0.087747	-2.905609	0.372052
11	6	0	1.950214	-1.331170	0.056674
12	1	0	2.673886	-2.127053	0.135242
13	6	0	2.377901	-0.051399	-0.110711
14	6	0	1.454353	1.010766	-0.247217
15	6	0	0.123268	0.754053	-0.277282
16	1	0	-1.581853	-4.179410	0.504655
17	1	0	-3.927785	-3.396244	0.178871
18	1	0	-4.633286	0.767931	-0.325656
19	1	0	0.768000	-3.689951	0.578646
20	1	0	1.792280	2.021690	-0.334220
21	6	0	-0.472492	2.156863	-0.555360
22	8	0	-0.833964	2.446352	-1.724745
23	9	0	-2.182852	1.465429	-0.341409
24	9	0	-5.742920	-1.585104	-0.131684
25	6	0	3.890012	0.236526	-0.157810
26	8	0	4.359277	1.377830	-0.307670
27	7	0	4.691036	-0.862778	-0.021844
28	6	0	5.124962	-1.312964	1.308547
29	1	0	4.267039	-1.552557	1.901390
30	1	0	5.742403	-2.180985	1.207528
31	1	0	5.681294	-0.533035	1.785103
32	6	0	5.141469	-1.625741	-1.194831
33	1	0	4.290807	-2.001476	-1.724073
34	1	0	5.708970	-0.987262	-1.839181
35	1	0	5.752496	-2.443838	-0.875045
36	7	0	-0.641388	3.133667	0.530102
37	6	0	-0.553869	4.443138	0.271128
38	1	0	-1.477066	4.786537	-0.146825
39	1	0	-0.354014	4.973612	1.178628
40	1	0	0.239477	4.616685	-0.425566
41	6	0	-0.906460	2.671493	1.900150
42	1	0	-0.458419	3.347929	2.597712
43	1	0	-1.962865	2.635633	2.066351
44	1	0	-0.491039	1.694504	2.033632

Equilibrium Geometry for Frequency Calculations of TS2 at M06-2X/6-311++G**

freq 6-311++g(d,p) scrf=(iefpcm,solvent=chloroform) geom=connectivity m062x

Number of imaginary frequencies = 1

Center Number	Atomic Number	Atomic Type	Coordinates (Angstroms) X	Y	Z
1	6	0	-4.577616	-1.283408	0.021040
2	6	0	-3.666681	-2.299685	0.054823
3	6	0	-2.293721	-2.000527	-0.036538
4	6	0	-1.810856	-0.662443	-0.158974
5	6	0	-2.837931	0.306282	-0.195156
6	6	0	-4.182161	0.040969	-0.107757
7	6	0	-1.380943	-3.105044	-0.010442
8	6	0	-0.358656	-0.417981	-0.243804
9	6	0	0.484600	-1.569502	-0.203150
10	6	0	-0.056128	-2.895182	-0.097135
11	6	0	1.886205	-1.465531	-0.245117
12	1	0	2.470705	-2.379213	-0.212296
13	6	0	2.504844	-0.243575	-0.338172
14	6	0	1.699115	0.895475	-0.413819
15	6	0	0.316769	0.837371	-0.350655
16	1	0	-1.787167	-4.105671	0.076867
17	1	0	-3.996232	-3.326595	0.146926
18	1	0	-4.898421	0.850405	-0.148247
19	1	0	0.639147	-3.726654	-0.080946
20	1	0	2.178308	1.861960	-0.527717
21	6	0	-0.248649	2.240020	-0.485202
22	8	0	-0.348974	2.742236	-1.592954
23	9	0	-2.535575	1.604441	-0.326597
24	9	0	-5.888638	-1.552511	0.109751
25	6	0	3.993028	-0.091155	-0.484478
26	8	0	4.437973	0.667006	-1.337334
27	7	0	4.793696	-0.848106	0.307552
28	6	0	4.398628	-1.446981	1.576465
29	1	0	3.380032	-1.173546	1.836890
30	1	0	4.480722	-2.536527	1.534478
31	1	0	5.064023	-1.078969	2.361960
32	6	0	6.228723	-0.809053	0.063046
33	1	0	6.414947	-0.676863	-0.999764
34	1	0	6.700121	0.013880	0.609729
35	1	0	6.662911	-1.751844	0.398162
36	7	0	-0.442704	2.931461	0.666481
37	6	0	-0.981316	4.279016	0.561053
38	1	0	-2.073906	4.255915	0.479004
39	1	0	-0.702479	4.840223	1.453195
40	1	0	-0.571931	4.765790	-0.320325
41	6	0	-0.651057	2.275186	1.947079
42	1	0	-0.251034	2.906017	2.742269
43	1	0	-1.719966	2.115099	2.131290
44	1	0	-0.137981	1.316283	1.975566

Equilibrium Geometry of Control Amide 8 at M06-2X/6-311++G (For Amide Isomerization)**

opt freq 6-311++g(d,p) scrf=(iefpcm,solvent=dmso) geom=connectivity m062x temperature=393.15

Center Number	Atomic Number	Atomic Type	Coordinates (Angstroms) X	Y	Z
1	6	0	-2.995270	0.680165	0.569445
2	6	0	-4.357455	0.773006	0.691277
3	6	0	-5.103412	-0.378111	0.446893
4	6	0	-4.521490	-1.581730	0.160558
5	6	0	-3.118241	-1.651462	0.054426
6	6	0	-2.487464	-2.934490	-0.091766
7	6	0	-1.142693	-3.046374	-0.064119
8	6	0	-0.300172	-1.881153	-0.041129
9	6	0	1.096487	-2.041944	-0.108967
10	6	0	1.930010	-0.950666	-0.189703
11	6	0	1.361207	0.326808	-0.313482
12	6	0	-0.006203	0.516460	-0.263095
13	6	0	-0.874494	-0.585124	-0.029879
14	6	0	-2.314019	-0.487321	0.171888
15	6	0	3.412783	-1.169467	-0.307050
16	6	0	5.666166	-0.441571	0.158957
17	6	0	3.828117	0.506125	1.495745
18	6	0	-0.504649	1.851764	-0.760787
19	6	0	0.559280	3.016997	1.150591
20	6	0	-0.370787	4.254961	-0.760386
21	7	0	4.228980	-0.330182	0.369061
22	7	0	0.004789	2.967224	-0.196032
23	8	0	3.839153	-2.065225	-1.032627
24	8	0	-1.259541	1.876952	-1.728566
25	1	0	-4.825946	1.696943	1.002525
26	1	0	-5.127785	-2.471751	0.049166
27	1	0	-3.119223	-3.811937	-0.165421
28	1	0	-0.666712	-4.019532	-0.104834
29	1	0	1.515665	-3.042182	-0.109750
30	1	0	2.004123	1.180647	-0.504641
31	1	0	6.109082	-1.159770	0.855799
32	1	0	5.862941	-0.767429	-0.859242
33	1	0	6.118139	0.536896	0.324434
34	1	0	4.493153	0.298382	2.337185
35	1	0	3.906409	1.566603	1.241633
36	1	0	2.810668	0.282027	1.805198
37	1	0	-0.070879	3.657228	1.774531
38	1	0	0.583980	2.025407	1.594322
39	1	0	1.570516	3.430849	1.130749
40	1	0	0.403499	4.981776	-0.513442
41	1	0	-0.460520	4.171324	-1.840613
42	1	0	-1.325687	4.598134	-0.348032
43	1	0	-2.433030	1.542901	0.860084
44	1	0	-6.167884	-0.310869	0.532205

Equilibrium Geometry for TS of Amide Isomerization of Control Amide 8 at M06-2X/6-311++G**

opt=(calcfc,ts,noeigentest) freq 6-311++g(d,p) scrf=(iefpcm,solvent=dmso) geom=connectivity m062x temperature=393.15

Number of imaginary frequencies = 1

Center Number	Atomic Number	Atomic Type	Coordinates (Angstroms) X	Y	Z
1	6	0	1.876448	-0.990355	-0.138228
2	1	0	1.412908	-3.052984	-0.416070
3	6	0	1.014972	-2.053898	-0.274746
4	6	0	-0.018371	0.531730	-0.117810
5	6	0	-0.381254	-1.867159	-0.229896
6	6	0	1.346492	0.310167	-0.103196
7	6	0	-0.919747	-0.572254	-0.042797
8	6	0	-1.256244	-2.988480	-0.439002
9	1	0	2.009724	1.166470	-0.124406
10	6	0	-2.598079	-2.832216	-0.449832
11	1	0	-0.809521	-3.958205	-0.627389
12	1	0	-3.255625	-3.666673	-0.663454
13	6	0	-3.188953	-1.574100	-0.086411
14	6	0	-4.587077	-1.481481	0.070344
15	6	0	-2.347975	-0.467856	0.199869
16	6	0	-5.117516	-0.329845	0.580796
17	1	0	-5.227083	-2.319316	-0.176040
18	6	0	-4.326153	0.738045	1.005766
19	1	0	-4.756169	1.602403	1.493646
20	6	0	-2.973078	0.630708	0.824094
21	6	0	-0.459959	1.905518	-0.518355
22	8	0	-1.465864	2.088649	-1.158868
23	7	0	0.444240	2.975878	-0.189036
24	6	0	1.038719	3.519015	-1.419210
25	1	0	1.775280	4.273558	-1.140571
26	1	0	0.284490	3.977688	-2.071584
27	1	0	1.545587	2.724272	-1.970061
28	6	0	-0.269244	4.029908	0.543590
29	1	0	0.453039	4.799464	0.818409
30	1	0	-0.702506	3.617166	1.454662
31	1	0	-1.064808	4.485504	-0.059474
32	6	0	3.357337	-1.240901	-0.214600
33	8	0	3.792414	-2.049937	-1.031522
34	7	0	4.163491	-0.523457	0.599000
35	6	0	5.605259	-0.654054	0.436433
36	1	0	5.995741	-1.473897	1.047139
37	1	0	6.075004	0.277515	0.753420
38	1	0	5.840564	-0.845401	-0.607433
39	6	0	3.735998	0.173327	1.807774
40	1	0	4.347299	-0.173143	2.644314
41	1	0	2.695701	-0.040958	2.036442
42	1	0	3.870162	1.252868	1.701626
43	1	0	-2.369172	1.407330	1.244874
44	1	0	-6.176574	-0.245210	0.707816
